# Discovery and Characterization of ZL-2201, a Potent, Highly Selective, and Orally Bioavailable Small-molecule DNA-PK Inhibitor

**DOI:** 10.1158/2767-9764.CRC-23-0304

**Published:** 2023-09-01

**Authors:** Shruti Lal, Neil E. Bhola, Bee-Chun Sun, Yuping Chen, Tom Huang, Vivian Morton, Kevin X. Chen, Shanghua Xia, Haoyu Zhang, Nehal S. Parikh, Qiuping Ye, O. Petter Veiby, David I. Bellovin, Yuhua Ji

**Affiliations:** 1 Biologics Discovery, Zai Lab (US) LLC, Menlo Park, California.; 2 WuXi AppTec, Shanghai, P.R. China.

## Abstract

DNA-dependent protein kinase (DNA-PK), a driver of the non-homologous end-joining (NHEJ) DNA damage response pathway, plays an instrumental role in repairing double-strand breaks (DSB) induced by DNA-damaging poisons. We evaluate ZL-2201, an orally bioavailable, highly potent, and selective pharmacologic inhibitor of DNA-PK activity, for the treatment of human cancerous malignancies. ZL-2201 demonstrated greater selectivity for DNA-PK and effectively inhibited DNA-PK autophosphorylation in a concentration- and time-dependent manner. Initial data suggested a potential correlation between ataxia-telangiectasia mutated (ATM) deficiency and ZL-2201 sensitivity. More so, ZL-2201 showed strong synergy with topoisomerase II inhibitors independent of ATM status *in vitro*. *In vivo* oral administration of ZL-2201 demonstrated dose-dependent antitumor activity in the NCI-H1703 xenograft model and significantly enhanced the activity of approved DNA-damaging agents in A549 and FaDu models. From a phosphoproteomic mass spectrometry screen, we identified and validated that ZL-2201 and PRKDC siRNA decreased Ser108 phosphorylation of MCM2, a key DNA replication factor. Collectively, we have characterized a potent and selective DNA-PK inhibitor with promising monotherapy and combinatory therapeutic potential with approved DNA-damaging agents. More importantly, we identified phospho-MCM2 (Ser108) as a potential proximal biomarker of DNA-PK inhibition that warrants further preclinical and clinical evaluation.

**Significance:**

ZL-2201, a potent and selective DNA-PK inhibitor, can target tumor models in combination with DNA DSB-inducing agents such as radiation or doxorubicin, with potential to improve recurrent therapies in the clinic.

## Introduction

Cells undergo numerous DNA insults that can lead to genomic instability, which is a hallmark of cancer. In response to these insults, DNA damage response (DDR) pathways are initiated to manage genomic instability through the detection of DNA lesions, which trigger the repair process (1, 2). DNA double-strand breaks (DSB) are considered the most deleterious form of DNA damage, and if left unrepaired, can lead to prolonged cell-cycle arrest and potential cell death (3). DDR often takes two main paths: homologous recombination (HR), which requires a sister chromatid template to complete an error-free DNA repair, or non-homologous end joining (NHEJ), which does not require a template, often resulting in error-prone repair (4, 5). Cancer cells utilize these repair processes to overcome genomic insults, proliferate, and acquire resistance to various therapeutic modalities. Therefore, targeting the DNA damage repair pathway is a promising approach to treat cancer and augment the efficacy of current standard-of-care therapeutics.

DNA-dependent protein kinase (DNA-PK), a serine and threonine kinase, belongs to the PI3K-related protein kinase (PIKK) family of proteins and plays a critical role in the NHEJ repair pathway (6, 7). In response to DNA damage, Ku-70 (encoded by *XRCC6*) and Ku-80 (encoded by *XRCC5*), bind to DSB ends. Following the recognition of DSBs and Ku binding, DNA-dependent protein kinase catalytic subunit (DNA-PKc), XRCC4-DNA ligase IV complex, XRCC4-like factor, and Artemis assemble at the damaged site, and DNA-PK kinase activity is triggered, followed by processing of the broken ends and dissociation of the NHEJ repair machinery (8, 9). DNA-PK autophosphorylation at multiple sites, including Ser2056, results in activation of DNA-PK activity and triggering of NHEJ-mediated DSB repair (10, 11).

DNA-PK activity is highly dysregulated in various cancers such as hepatocellular carcinoma, melanoma, and other solid tumors (12, 13). Increased DNA-PK expression is also associated with resistance to chemotherapeutic agents and reduced radiosensitization (14–18). In addition, while HR and alternative end joining is restricted to S- and G_2_-phases of the cell cycle, DNA-PK–driven NHEJ occurs across all phases of the cell cycle. Specifically, several lines of experimental evidence show that cells that lack DNA-PK activity or have pharmacological inhibition of DNA-PK fail to engage in the NHEJ pathway and can effectively sensitize cells to current standard-of-care treatments such as ionizing radiation (IR) or other DSB-inducing chemotherapeutic agents (19–21). Several DNA-PK inhibitors have been identified, such as NU7441, NU7026, and KU-0060648, but they have demonstrated very poor selectivity for DNA-PK (21–23). Other DNA-PK inhibitors such as CC-115 target both DNA-PK and the mTOR, another PIKK family member (24). The newer generation of DNA-PK inhibitors, such as NU5455, M3814, and AZD7648, are more selective for DNA-PK and have progressed to clinical trials in combination with radiotherapy and liposomal doxorubicin, respectively (NCT02516813 and NCT03907969) in patients with advanced malignancies (19, 20). Studies have shown that mutations in DDR-related genes, such as loss of function of ataxia-telangiectasia mutated (*ATM*), a key player in HR repair, or MutS homolog 3 (*MSH3*), a mismatch repair protein, have been shown to increase cellular sensitivity to DNA-PK inhibitors and may serve as predicitve biomarkers (25–27).

In this article, we describe ZL-2201, an orally bioavailable, highly potent, and selective pharmacologic inhibitor of DNA-PK for the treatment of cancer. We interrogated its *in vitro* activity in ATM-deficient lung cancer models and demonstrated its synergistic antiproliferative activity with different classes of compounds used in cancer treatment. ZL-2201 treatment *in vivo* enhances antitumor efficacy in combination with the topoisomerase II inhibitor doxorubicin, as well as IR. Finally, a mass spectrometry (MS) screen identified new phosphoproteins that were altered upon ZL-2201 treatment to provide initial mechanistic insight into DNA-PK inhibition as clinical development continues.

## Materials and Methods

### Chemistry

Synthesis of ZL-2201: The chemical and synthesis details are described in the Supplementary Data (Supplementary Fig. S1).

### Cell Lines

Normal human colon epithelial cells (nHCEC; H-6047) were purchased from Cell Biologics and maintained in complete epithelial cell medium kit (H6621) while all other cells were purchased from ATCC at the beginning of the study in 2021. NCI-H1703 (RRID: CVCL_1490), NCI-H522 (RRID: CVCL_1567), NCI-H1373 (RRID: CVCL_1465), NCI-H1395 (RRID: CVCL_1467), NCI-H23 (RRID: CVCL_1547) were cultured in RPMI1640 supplemented with 10% FBS, 1% penicillin-streptomycin, and 1% l-glutamine. A549 (RRID: CVCL_0023) was cultured in F-12K media supplemented with 10% FBS, 1% Pen-Strep, and 1% l-glutamine. M059J (RRID: CVCL_0400) and M059K (RRID: CVCL_0401) cells were cultured in DMEM/F12 media supplemented with 10%FBS, 1% Penn-Strep, and 1% l-glutamine. MDCK (RRID: CVCL_0422) and FaDu (RRID: CVCL_1218) cells were cultured in Eagle Minimum Essential Medium supplemented with 10% FBS, 1% Penn-Strep, and 1% l-glutamine. A549 ATM knockout (KO; ab276095) cell line was bought from Abcam and Fadu ATM KO cells were created by Wuxi AppTec using CRISPR. Cells were cultured in a contamination-free environment utilizing the vendor-recommended media and several vials were frozen down from passages 1 and 2. All experiments were performed in early passaged cells (passage number between 4–9; cells were passaged when they have achieved 70%–80% confluence). Cells were discarded after the 9th passage and a new vial was thawed. *Mycoplasma* testing was not conducted on any cell lines.

### DNA-PK and Other Kinases Assay

ZL-2201 was tested against selected kinases using the Eurofins standard KinaseProfiler assays and following the relevant standard operating procedures. Lipid kinases, ATM(h), ATR/ATRIP(h), and DNA-PK(h) were assayed using an homogeneous time-resolved fluorescence (HTRF) format, whereas other protein kinases were assayed in a radiometric format. General information regarding this screening assay is available on the Eurofins website: http://www.eurofins.com/pharmadiscovery. Briefly, a working stock of 50x final assay concentration in 100% DMSO was prepared from a 10 mmol/L stock solution in DMSO. The required volume of the 50x stock of test compound was added to the assay well before a reaction mix containing the enzyme and substrate was added. The reaction was initiated by the addition of ATP at the selected concentration. There was no preincubation of the compound with the enzyme/substrate mix prior to ATP addition. Full details of the assay for each kinase are available on the Eurofins website. Data are handled using a custom built in-house analysis software (Eurofin). Results are expressed as kinase activity remaining, as a percentage of the DMSO control. This is calculated using the following formula:



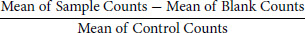



For IC_50_ determinations, data are analyzed using XLFit version 5.3 (ID Business Solutions). Sigmoidal dose–response (variable slope) curves are fit based on the mean result for each test concentration using nonlinear regression analysis.

### Pharmacokinetic Analysis for ZL-2201

Following single oral administration of ZL-2201 at 15 and 60 mg/kg to Balb/c nude mice, plasma samples were collected at the timepoints of 0.5, 1, 1.5, 2, 4, 6,8, 12, 18, and 24 hours (*n* = 3) after dose. Following single oral or intravenous administration of ZL-2201 at the doses indicated in Table 2 to CD-1 mice, sprague-dawley (SD) rats, beagle dogs, and cynomolgus monkeys, plasma samples were collected at the timepoints of 0.0833, 0.25, 0.5, 1, 2, 4, 8, 12, and 24 hours (*n* = 3) after dose for intravenous, and 0.25, 0.5, 1, 2, 4, 8, 12, and 24 hours after dose for oral administration. The plasma samples were stored at −80°C until analysis. The analytic standards spiked with ZL-2201 and the plasma samples were cleaned by protein precipitation and analyzed for the concentration of ZL-2201 on a LC/MS-MS system (Applied Biosystems) with standard curves over the range of 2 to 6,000 nmol/L. The plasma concentration–time profiles of ZL-2201 were used to estimate the pharmacokinetic parameters with Phoenix WinNonlin software (version 8.2, Certara).

### Human Pharmacokinetic Prediction

The human clearance of ZL-2201 was predicted on the basis of the predicted clearances with hepatocyte *in vitro*-*in vivo* correlation (IVIVC) method and allometric scaling from preclinical data. The human volume distribution was predicted with Øie-Tozer method and allometric scaling from preclinical data. Following were the main assumptions for human pharmacokinetic profile projection:

- Mean predicted human pharmacokinetic parameters for the simulation: CL = 0.360 L/hour/kg; Vss = 1.54 L/kg; fu = 0.491; F = 70%
- Linear pharmacokinetic in human over the dose ranges
- No change in parameters including CL and Vss upon repeated dosing
- Human body weight 60 kg

### Cell-cycle Assay

Cells were seeded in 6-well plates at the cell density of 1 million cells per well with 3 mL of media per well. Cells were cultured overnight. The next day, cells were treated with doxorubicin (0.006 μmol/L), ZL-2201 (0.3 μmol/L and 3.6 μmol/L), or a combination of doxorubicin and ZL-2201 for 72 hours. Cells were collected in a falcon tube, washed in PBS, and fixed by adding 70% cold ethanol dropwise with continuous slow vortexing and incubated at −20°C for at least 24 hours. The cells were then stained with propidium iodide (PI) supplemented with RNase (Thermo Fisher Scientific; F10797) at room temperature for 15 minutes in dark. DNA content of cells was evaluated by flow cytometry using an LSRFortessa (BD Biosciences) and analyzed using FlowJo software (RRID:SCR_008520; BD Biosciences).

### Simple Western Assay

Cells were seeded in 6-well plates at a density of 1–2 million cells per well. The next day, the medium was removed, and the cells were stimulated with bleomycin (10 μmol/L; Cayman Chemicals; NC1655250) or doxorubicin (0.006 μmol/L; Thermo Fisher Scientific; AAJ64000MF) in fresh medium. ZL-2201 was added at various indicated concentrations and times. Untreated cells were used as controls. After the completion of each incubation period, the cells were washed once with PBS and harvested using RIPA cell lysis buffer (Thermo Fisher Scientific; PI89900) supplemented with a 3X Halt Protease Phosphatase cocktail (Thermo Fisher Scientific; PI78445). The cells were then centrifuged at 12,000 rcf for 20 minutes at 4°C. The supernatant containing the proteins was collected and transferred to a new tube. Proteins were stored at −20°C. Protein concentration was determined using a bicinchoninic acid (BCA) protein assay kit (Thermo Fisher Scientific; PIA53226) following the manufacturer's protocol. Simple Western Jess plates (SM-W012, SM-W004, or SM-W007) were set up, depending on the molecular size of the protein of interest, following the manufacturer's instructions. The data were analyzed using ProteinSimple Compass software. The following antibodies were used: pDNA-PK (RRID:AB_2939025; Cell Signaling Technology; CST68716), DNA-PK (RRID:AB_731982; Abcam; Ab44815), γH2AX (RRID:AB_2118010; Cell Signaling Technology; CST2577), H2AX (RRID:AB_2118009; Cell Signaling Technology; CST9718), GAPDH (RRID:AB_2278695; R&D Systems; AF5718), pMCM2 (S108; RRID:AB_242513; Bethyl Lab; A300-094A), vinculin (RRID:AB_10559207; Cell Signaling Technology; CST4650), rabbit anti-goat IgG-HRP (R&D Systems; HAF017), and goat anti-rabbit IgG (H+L; Jackson ImmunoResearch; 111-035-045).

### Cell Titer Glo Cellular Cytotoxicity Assay

Cells were seeded in 384-well plates at a density of 300–500 cells per well (depending upon the doubling time for each cell line) with 40 μL of media per well using a multidrop combi cell dispenser (Thermo Fisher Scientific). The cells were incubated at room temperature for 30 minutes to minimize edge effects. The next day, the cells were treated with drugs of interest at various concentrations using a D300e digital dispenser (Tecan). Following treatment, the cells were cultured at 37°C for 6 days. On day 6 after treatment initiation, the plates and Cell Titer Glo (CTG; Promega; PRG9243) were incubated at room temperature (protected from light) for 1 hour to reach equilibrium. After incubation, half of the CTG volume was added to one volume of cell culture medium in each well. The plates were then shaken for 30 seconds to induce cell lysis. The cells were then incubated at room temperature for 12 minutes protected from light to stabilize the luminescence signal. The luminescent cell viability was measured using an EnVision plate reader (PerkinElmer). For cell viability, the data were analyzed using Excel (Microsoft) and GraphPad Prism (RRID:SCR_002798; Dotmatics). The data were first transformed to a log scale on the *X*-axis, then transformed values were normalized, and finally, normalized values were fitted using a nonlinear regression curve fit. For synergy, the data were analyzed using Excel, SynergyFinder (28), and Combenefit (29). The Bliss, Loewe, and HSA models were used to determine the synergy between the drugs and ZL-2201 inhibitory effect. The formula used was % inhibition = 100 × [1 − (X − MIN)/(MAX − MIN)]. The D300e Control software was used to design the titration and synergy matrices.

### High-content Imaging Assay

Cells were seeded in 96-well plates at a density of 10,000 cells per well with 100 μL of media per well. The cells were cultured overnight. The next day, the cells were treated with doxorubicin (0.006 μmol/L), ZL-2201 (0.1, 0.3, 0.5, 1, and 3.6 μmol/L), and a combination of doxorubicin and ZL-2201 for the indicated times. The cells were washed in PBS and fixed by adding Cytofix/Perm buffer (BD; 554714) at room temperature for 20 minutes. Cells were washed with Perm wash buffer and blocked with Perm wash buffer and 3% FBS at room temperature for 1 hour. The cells were then incubated with primary antibodies against γH2AX-Ser139 (RRID:AB_309864; Millipore; 05-636), pPH3 (Ser10; 6G3; RRID:AB_331748; Cell Signaling Technology; 9706) and cleaved caspase 3 (Asp175; 5A1E; RRID:AB_2070042; Cell Signaling Technology; 9664) overnight at 4°C. Cells were washed the following day and incubated with secondary antibodies (Alexa Fluor 488; Invitrogen; A32723 or A32731 and Alexa Fluor 647; A32733) at room temperature for 45 minutes in the dark. The cells were washed and stained with Hoescht (Thermo Fisher Scientific; H3570) at room temperature for 15 minutes in the dark. Images were obtained and quantified using the ImageXpress Micro Confocal High-Content Imaging System (Molecular Devices). A total of 16 fields were obtained per image for each timepoint and treatment groups.

### Phospho-MS Screen

NCI-H1703 cells were seeded in duplicate 10 cm plates and allowed to proliferate for 48 hours from the following day. One set of duplicates were transfected with 145 pmol of PRKDC siRNA. After 48 hours, plates were treated with 300 nmol/L ZL-2201 for 4 and 24 hours followed by 10 μmol/L Bleomycin treatment for 3 hours. Cell pellets were collected after PBS washing and a small aliquot was obtained for immunoblot validation. Cell pellets were flash frozen and sent to MSBioworks LLC. Cell pellets were lysed in 400 μL urea buffer (8 mol/L urea, 50 mmol/L Tris-HCl, 1X protease/phosphatase inhibitors), incubated for 1 hour at room temperature and quantified by Qubit. A total of 500 μg of samples were diluted in 25 mmol/L ammonium bicarbonate, reduced, and alkylated followed by trypsin digestion overnight. Peptides were quenched with formic acid followed by lyophilization. Peptides were resuspended and enriched using TiO2 enrichment kit (GL Sciences). Peptides were analyzed by nano LC/MS-MS using HPLC system interfaced to a Thermo Fisher Scientific Fusion Lumos. Data was processed using MaxQuant software.

### *In Vivo* Studies

#### Tumor Inoculation

The NCI-H1703 human lung cancer cells were collected by trypsinization and prepared in a 1:1 mixture of sterile PBS and Matrigel. Female Balb/c nude mice were purchased from Charles River at 6–8 weeks of age were inoculated subcutaneously on the right flank with 5 × 10^6^ tumor cells. All *in vivo* studies for animal testing and research at ZaiLab were performed in accordance to an approved Institutional Animal Care and Use Committee protocol. A549 and FaDu xenograft studies were performed at WuXi AppTec. 5 × 10^6^ cells were inoculated in female Balb/c nude mice at 6–8 weeks old on the right hind flank. The protocol and any amendment(s) or procedures involving the care and use of animals in these studies were reviewed and approved by the Institutional Animal Care and Use Committee (IACUC) of WuXi AppTec. During the studies, the care and use of animals will be conducted in accordance with the regulations of the Association for Assessment and Accreditation of Laboratory Animal Care (AAALAC).

#### Treatments

Tumor-bearing mice were observed twice per week until the average tumor volume was approximately 100 mm^3^. Mice were then randomized into treatment groups such that the average volume and variance were similar. Tumor-bearing mice were administered ZL-2201 by oral gavage (orally) at the dose levels of 5, 15, 30, or 60 mg/kg. Control group was given vehicle [0.5% hydroxypropyl methylcellulose/0.2% Tween-80] only. Each treatment group consisted of *n* = 8–10 tumor-bearing mice. Combination *in vivo* studies with pegylated liposomal doxorubicin (PLD) and IR were performed by WuXi Apptec. PLD was administered at a dose of 2.5 mg/kg once weekly. Focused IR was administered at 2 Gy.

#### Tissue Collection

Following ZL-2201 treatment, tumor-bearing mice were euthanized by cardiac puncture. Timepoints of collection were 0.5, 1, 1.5, 2, 4, 6, 8, 12, 18, and 24 hours after single dose administration. Blood was collected into tubes containing Ethylenediaminetetraacetic acid (EDTA) and mixed to inhibit coagulation. Tubes were maintained at 4°C on ice and then centrifuged at 2,000 rpm at 4°C in a refrigerated centrifuge. Plasma was transferred into a 96-well plate and stored at −20°C until analyzed for ZL-2201 levels. Tumors were collected by surgical removal after euthanasia. The overlying skin was removed, and the tumor tissue was placed into 2 mL screw-top tubes. Tubes were placed into liquid nitrogen for snap freezing and subsequently transferred to a −80°C freezer for storage until analysis.

#### Sample Preparation for Simple Western Assay

Tumor tissues were weighed and cut into 20 mg pieces on ice. The tissues were transferred to prechilled 2 mL benchmark tubes containing zirconium beads. Cold RIPA (300 μL) buffer along with Halt protease and phosphatase inhibitors (3X) were added per 20 mg of tumor tissues. The tubes were transferred to a BeadBlaster microtube homogenizer (Benchmark). The tumor tissues were homogenized at the following settings: 6.0 (m/s speed); 15 seconds; 3 cycles; 30-second m/s interruption. The supernatant was transferred to new prechilled microcentrifuge tubes and centrifuged at 12,000 rcf for 20 minutes at 4°C. The supernatant containing protein was collected and transferred to a new chilled microcentrifuge tube. Proteins were stored at −20°C. Protein concentration was determined by the BCA protein assay kit and Simple Western Jess plates were prepared following the manufacturer's protocol.

### Statistical Analysis

*In vivo* data were analyzed using either a one-way ANOVA or a Kruskal–Wallis test to determine which groups were significantly different from one another on the final day of measurement. One-way ANOVA was used to calculate the significance between the vehicle group and treatment groups. Kruskal–Wallis test was used to determine the statistical significance between the combination group and single treatment groups. IC_50_ calculations and statistical comparisons were made using GraphPad Prism software.

### Data Availability

All relevant data supporting the findings in this study are within the article and its Supplementary Data and from the corresponding author within reasonable request.

## Results

### ZL-2201 is a Highly Selective and Potent Small-molecule Inhibitor of DNA-PK Activity

**FIGURE 1:**
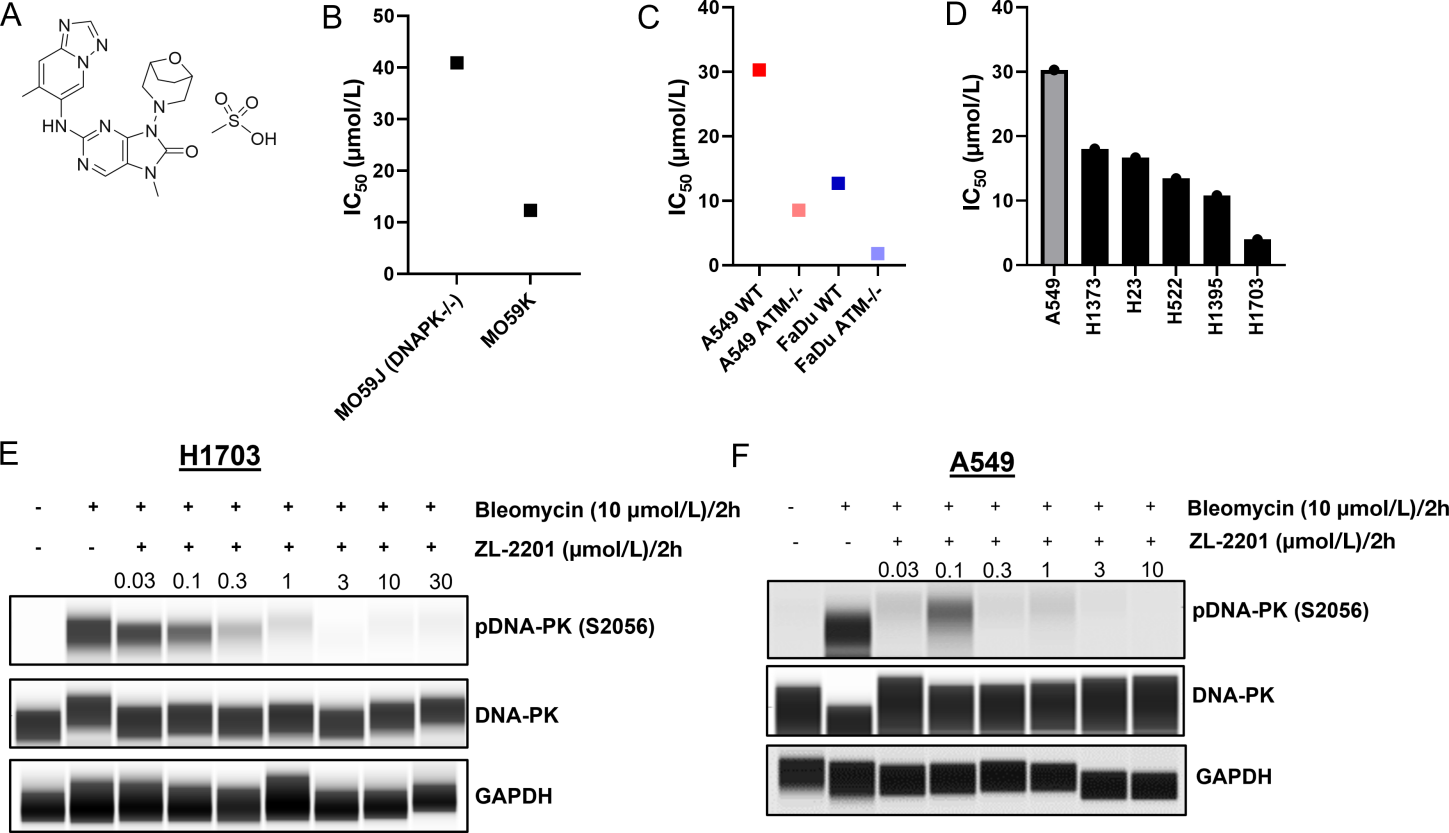
Evaluation of ZL-2201 antiproliferative activity *in vitro*. **A,** Chemical structure of ZL-2201, a potent and selective DNA-PK inhibitor. **B,** Concentration-dependent response to ZL-2201 in M059J and M059K glioblastoma cancer cells measured by CTG after 6-day of treatment. The graph represents the average inhibitory (IC_50_) values (*n* = 3). **C,** Concentration-dependent response to ZL-2201 in CRISPR KO of ATM in A549 and FaDu cancer cells measured by CTG after 6 days of treatment. The graph represents the average inhibitory (IC_50_) values (*n* = 2–5). **D,** IC_50_ responses to ZL-2201 in ATM mutant cell lines (black bars) versus ATM WT A549 cell line (gray bar). Cell growth was measured by CTG after 6 days of treatment. The graph represents the average inhibitory (IC_50_) values (*n* = 2–6). NCI-H1703 (**E**) and A549 (**F**) cancer cells were treated with bleomycin (10 μmol/L) for 2 hours followed by the addition of increasing concentration of ZL-2201 for 2 hours. Whole-cell lysates were harvested, and concentration-dependent inhibition of DNA-PK protein were analyzed by Simple Western.

To evaluate the pharmacologic effect of DNA-PK inhibition in tumor models, the DNA-PK kinase inhibitor ZL-2201 was developed (Fig. 1A; Supplementary Fig. S1) the ability of ZL-2201 to inhibit the enzymatic activity of DNA-PK was examined *in vitro*. Utilizing a HTRF kinase activity assay, the potency of ZL-2201 against DNA-PK was determined to be 0.4–0.9 nmol/L (0.5667 ± 0.2887 nmol/L) across three independent experiments. To further understand the selectivity of ZL-2201 over the most closely related kinases, the *in vitro* inhibition of other PI3K-related kinases (PIKK) was examined (Table 1). The activity of ZL-2201 against human mTOR, ATM, PI3Kα, PI3Kβ, PI3Kγ, and PI3Kδ PI3-kinases was assessed using HTRF (for DNA-PK and ATM) and standard radiometric approaches (for mTOR and PI3 kinase). The IC_50_ values for ZL-2201 were >1,000 nmol/L for all PI3 kinases tested, 577 nmol/L for mTOR, and 451 nmol/L for ATM. Next, we evaluated the cellular specificity of ZL-2201 for DNA-PK using the DNA-PK proficient and deficient cell line pair, MO59K and MO59J, respectively. We observed a 4-fold higher IC_50_ in MO59J (DNA-PK deficient) compared with MO59K (DNA-PK proficient) with ZL-2201 (Fig. 1B). These biochemical and cellular observations indicate that ZL-2201 is a DNA-PK selective inhibitor.

**TABLE 1:** *In vitro* pharmacology of ZL-2201 against PIKK family members

Enzyme	IC_50_ (nmol/L)	Selectivity ratio
DNA-PK	1.1	—
ATM	451	410
ATR	1,000	909
mTOR	577	577
PI3Kα	1,900	1,727
PI3Kβ	>10,000	>9.091
PI3Kγ	1,600	1,455
PI3Kδ	4,200	3,818

Abbreviations: ATM, ataxia-telangiectasia mutated; ATR, ATM and rad3-related protein kinase; DNA-PK, DNA-dependent protein kinase; IC_50_, half-maximal inhibitory concentration; mTOR, mammalian target of rapamycin; PI3K, phosphoinositide 3-kinase.

### ZL-2201 Decreases DNA-PK Phosphorylation and *In Vitro* Proliferation of Non–small Cell Lung Cancer Cell Lines

To further characterize ZL-2201, we investigated the antiproliferative effect of ZL-2201. It has been shown that ATM loss is synthetic lethal with DNA-PK (30, 31) and more than 20% of non–small cell lung cancer (NSCLC) cell lines are ATM deficient (32, 33). First, we investigated the viability effect of ZL-2201 in A549 and FaDu ATM KO isogenic pair cell lines. A549 and FaDu ATM KO cell lines displayed 3.5- and 7-fold lower IC_50_s compared with their respective parental counterparts (Fig. 1C). Second, we evaluated the response of five ATM-deficient NSCLC cell lines compared with the ATM-proficient A549 cell line. The ATM mutational status and variant allele frequency was confirmed from Cancer Cell Line Encyclopedia data (RRID:SCR_014555; cbioportal.org) and Cell Model Passports (cellmodelpassports.sanger.ac.uk; Supplementary Table S1). A549 displayed a mean IC_50_ of 30.3 μmol/L while the ATM-deficient cell lines (H1373, H23, H522, H1395, H1703) displayed lower IC_50_ values of 18.1, 16.7, 13.5, 10.8, and 3.98 μmol/L, respectively (Fig. 1D; Supplementary Fig. S2; Supplementary Table S1). To orchestrate the response to DNA DSBs, autophosphorylation of DNA-PK at Ser2056 is critical for the activity of the NHEJ pathway (10, 11). We investigated the ability of ZL-2201 to selectively inhibit DNA-PK autophosphorylation at Ser2056 following treatment with the radiomimetic chemical, bleomycin. In both NCI-H1703 and A549 cell lines, bleomycin-induced DNA-PK phosphorylation was robustly diminished starting from 300 nmol/L ZL-2201 (Fig. 1E and F). Moreover, treatment with ZL-2201 prior to bleomycin displayed more robust time- and concentration-dependent decreases in DNA-PK phosphorylation (Supplementary Fig. S3). Collectively, these results confirm that ZL-2201 selectively inhibits DNA-PK autophosphorylation in NSCLC cells. Furthermore, initial evalaution of single-agent ZL-2201 activity suggests that ATM deficiency may contribute to increased antiproliferative sensitivity.

### ZL-2201 Demonstrates Target Engagement and Tumor Growth Inhibition *In Vivo*

**FIGURE 2:**
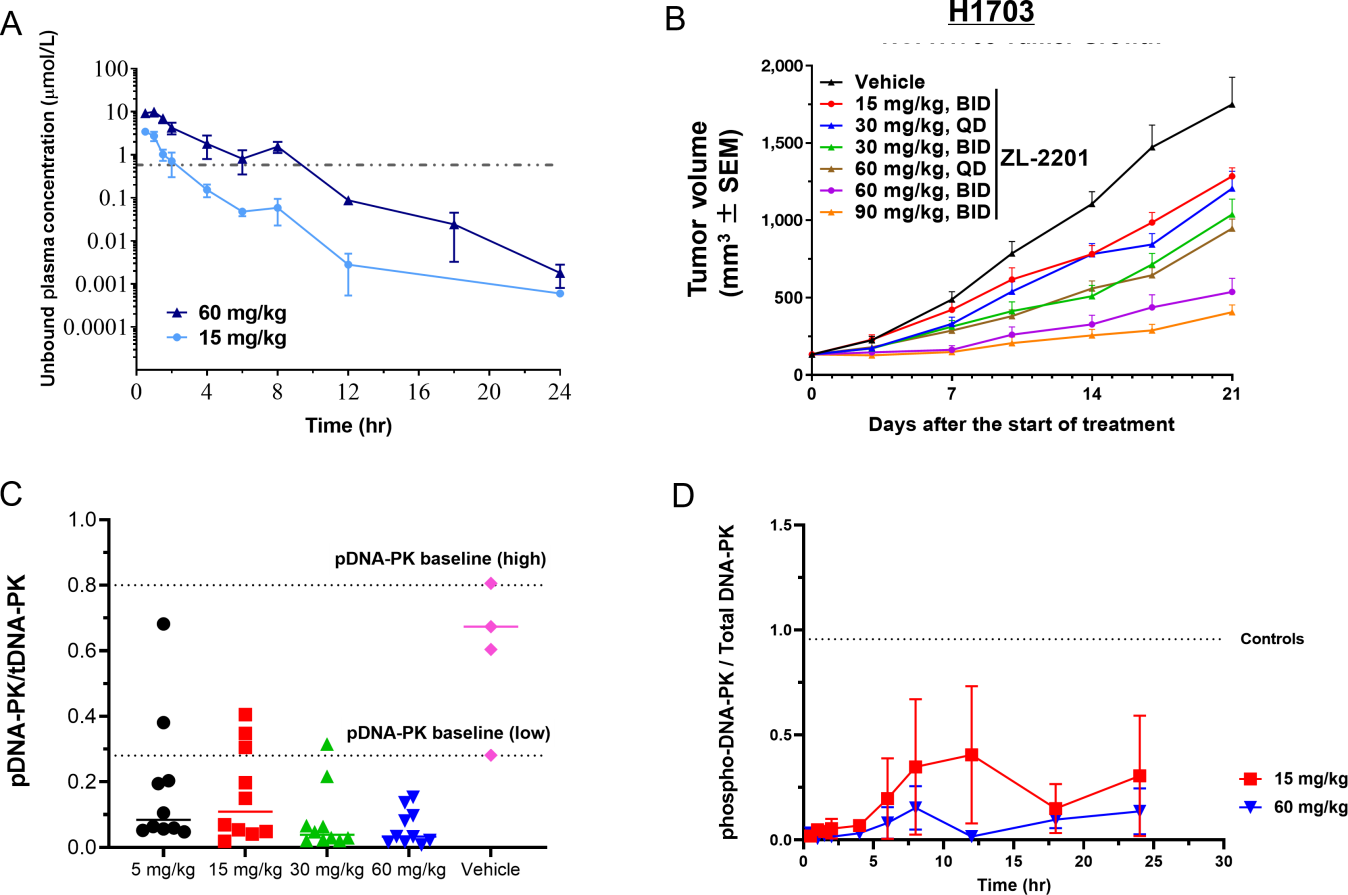
ZL-2201 inhibits DNA-PK autophosphorylation and exhibits potent antitumor efficacy *in vivo*. **A,** Unbound plasma concentration of ZL-2201 across multiple timepoints following one-time treatment with 15 and 60 mg/kg of ZL-2201 in Balb/c nude mice (*n* = 2–3). **B,** Average tumor volume of NCI-H1703 xenografts established in female Balb/c nude mice (*n* = 8–10/group) treated with various concentrations of ZL-2201 for a period of 21 days. QD: once a day; BID: twice a day. **C,** Western blot analysis of phospho-DNA-PK in NCI-H1703 mouse xenografts. Tumor tissues were harvested after every 2 hours over the course of 24 hours after the final dose. The ratio of pDNA-PK to total DNA-PK was determined in xenografts at baseline (no treatment) and after treatment with 5, 15, 30, and 60 mg/kg of ZL-2201. Individual timepoints were combined for each treatment group to show the ratio of pDNA-PK to total DNA-PK. **D,** Simple Western showing the pDNA-PK reduction after 15 and 60 mg/kg dose treatment in NCI-H1703 mouse xenografts collected at multiple timepoints after the final dose (*n* = 3).

Before, we interrogated the mechanism of action of ZL-2201, we sought to confirm that *in vitro* antiproliferative activity translated to *in vivo* tumor growth inhibition. ZL-2201 demonstrated high clearance, good oral bioavailability, and adequate pharmacokinetics in multiple preclinical species including mice (Fig. 2A; Table 2). Next, we treated NCI-H1703 tumor-bearing mice with different doses and scheduling regimens of ZL-2201. ZL-2201 resulted in significant dose-dependent tumor growth inhibition with near 100% tumor growth inhibiton (TGI) observed at 60 and 90 mg/kg twice daily (Fig. 2B; Supplementary Fig. S4A). ZL-2201 at all doses other than 90 mg/kg twice daily was well tolerated by tumor-bearing animals (Supplementary Fig. S4B). Two mice in the ZL-2201 90 mg/kg treatment group had a body weight loss of 10%–15% on day 8 after the start of treatment; however, no gross abnormalities were observed at full necropsy of these animals*.* Similar to our observations *in vitro*, xenograft samples collected after treatment displayed suppressed phosphorylated DNA-PK levels (Fig. 2C). Importantly, similar antitumor activity was observed at 60 mg/kg once daily, 30 mg/kg twice daily, 30 mg/kg once daily, and 15 mg/kg twice daily, suggesting that total drug exposure may be the most important characteristic for antitumor efficacy *in vivo.* This was further confirmed by phospho-DNA-PK immunoblot data collected from xenografts obtained at different timepoints following treatment with 15 and 60 mg/kg. Although not statistically significant, 15 mg/kg ZL-2201 displayed a rebound in phospho-DNA-PK levels after 12 hours while 60 mg/kg ZL-2201 maintained complete suppression of phospho-DNA-PK levels (Fig. 2D). The cumulative findings in this section indicate that increased exposure of ZL-2201 results in monotherapy activity *in vivo* and decreasing DNA-PK phosphorylation induced by endogenous DSBs.

**TABLE 2:** Drug metabolism and pharmacokinetics (DMPK) properties of ZL-2201 in preclinical species

		IV (*n* = 3, male)			PO (*n* = 3, male)					
Species	Dose IV/PO (mg/kg)	CL (L/hour/kg)	Vss (L/kg)	T_1/2_ (hours)	AUC_0-inf_ (mmol/L*hours)	C_max_ (mmol/L)	T_max_ (hours)	F (%)	Hepatocyte Cl_int_ (*n* = 2) (μL/minute/10^6^ cells)	PPB Fu (%)
Mouse	2.0/10.0	1.42 ± 0.0791	1.10 ± 0.0355	0.73 ± 0.085	23.2 ± 0.704	11.4 ± 1.27	0.5–1.0	134	NA	0.414
Rat	1.0/2.0	0.666 ± 0.0714	1.45 ± 0.0800	2.0 ± 0.06	5.65 ± 0.928	1.80 ± 0.928	0.5	76.2	3.6	0.455
Dog	1.0/1.0	0.415 ± 0.0408	1.93 ± 0.110	3.6 ± 0.20	6.40 ± 1.32	1.30 ± 0.145	0.25–1.0	108	0.84	0.570
Cyno	1.0/5.0	1.01 ± 0.124	1.50 ± 0.260	3.6 ± 3.8	14.0 ± 2.43	4.50 ± 0.750	0.5–2.0	115	5.1	0.560
Human (predicted)	<15 mg	0.360	1.54	2.8	NA	NA	NA	∼70	2.7	0.491

Abbreviations: AUC_0-inf_, area under the plasma concentration–time curve from time zero to infinity; CL, clearance; CL_int_, intrinsic clearance; C_max_, maximum plasma concentration; F, bioavailability (%) was calculated with mean AUC_0-inf_ and nominal dose; Fu, fraction unbound; IV, intravenous; NA, no analysis; PO, oral; PPB, plasma protein binding; T_1/2_, elimination half-life; T_max_, time to reach maximum plasma concentration; Vss, volume of distribution at steady state.

### ZL-2201 Synergizes with DSB-inducing Agents

**FIGURE 3:**
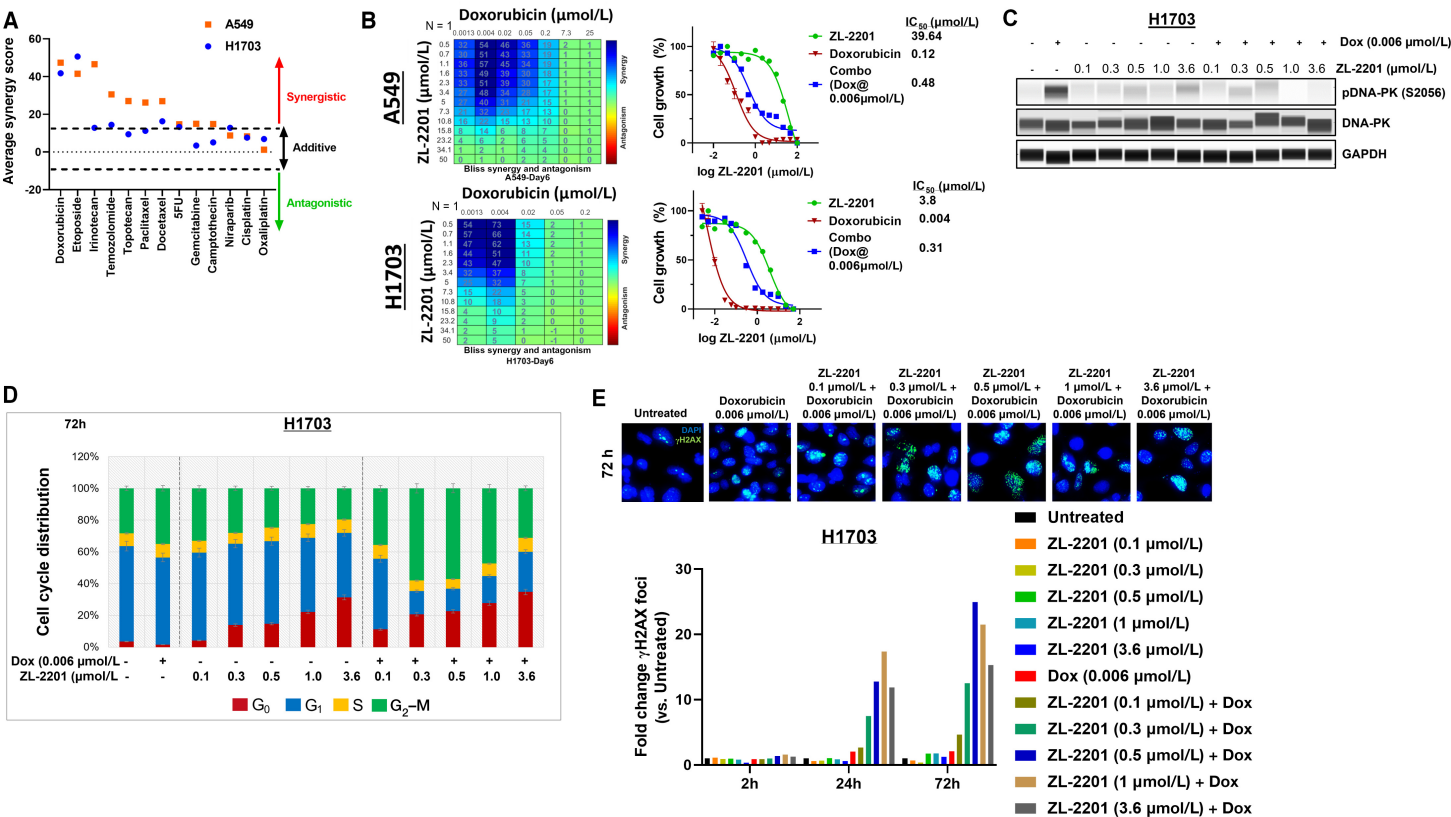
Phenotypic evaluation of ZL-2201 synergy with various DNA-damaging agents. **A,** Synergy scores of ZL-2201 in combination with various classes of antineoplastic drugs in A549 and NCI-H1703 cancer cells were analyzed after 6 days of combination treatments. The Bliss synergy score was measured using Combenefit and SynergyFinder. A synergy score of >10 is indicative of synergistic activity, <10 and >−10 are indicative of additive activity, and those below −10 are indicative of antagonism (*n* = 2–6). **B,** A549 and NCI-H1703 cells were cotreated with the increasing concentration of ZL-2201 and doxorubicin for 6 days. The synergistic activity of ZL-2201 with doxorubicin was analyzed by Combenefit. The blue color highlights the synergy. The graphs show the concentration-dependent cellular efficacy as a single agent as well as combinational treatments. One of the representative experiments is shown (*n* = 3). **C,** NCI-H1703 cells were treated with ZL-2201 (0.1, 0.3, 0.5, 1, and 3.6 μmol/L) and a low dose of doxorubicin (0.006 μmol/L) for 2 hours. Whole-cell lysates were harvested, and Simple Western was used to show the inhibition of pDNA-PK upon combination treatment of ZL-2201 with doxorubicin. **D,** NCI-H1703 cells were treated with ZL-2201 (0.1, 0.3, 0.5, 1 and 3.6 μmol/L) and a low dose of doxorubicin (0.006 μmol/L) for 72 hours. Single cells were harvested and stained with PI/RNAse solution. The cell-cycle distribution based on DNA content was determined by flow cytometry. **E,** NCI-H1703 cells were treated with ZL-2201 (0.1, 0.3, 0.5, 1, and 3.6 μmol/L) and a low dose of doxorubicin (0.006 μmol/L) for 2, 24, and 72 hours. Cells were stained with γH2AX (a DNA damage marker), and high-content imaging analysis was performed. One of the representative images of 72-hour timepoint is shown. Average yH2AX foci intensity across 16 fields/well was quantified. Blue: Hoescht; Green: γH2AX; Number of fields per image: 16.

We previously illustrated that the DSB-inducing radiomimetic agent bleomycin robustly increased DNA-PK phosphorylation. In addition, DNA-PK inhibition has been shown to improve the efficacy of DNA-damaging agents (19, 20), therefore we investigated the synergistic potential of ZL-2201 with several classes of DNA-damaging agents in both A549 and NCI-H1703 cell lines (Fig. 3A). The combination of ZL-2201 and doxorubicin showed strong synergy (synergy score >30) in both A549 and NCI-H1703 cells (Fig. 3A and B). Synergy with doxorubicin was also observed in four additional NSCLC cell lines (Supplementary Fig. S5A). Similar to doxorubicin, Etoposide, a topoisomerase II inhibitor, showed strong synergy (synergy score >30) with ZL-2201 in both A549 and NCI-H1703 (Fig. 3A). Good synergy with ZL-2201 (synergy score>10 and <30) was observed only in A549 cells treated with temozolomide, 5-fluorouracil, and the two topoisomerase I inhibitors, topotecan and irinotecan. Mild synergy (synergy scores >10 and <20) was observed with niraparib (PARP inhibitor), camptothecin (topoisomerase I inhibitor) gemcitabine, paclitaxel and docetaxel and addivity (synergy score >0 and <10) was observed with cisplatin and oxaliplatin (Fig. 3A).

Confirmation of doxorubicin induced DNA-PK phosphorylation was observed which was decreased with concentrations of ZL-2201 ranging from 100 nmol/L to 3.6 μmol/L (Fig. 3C). Furthermore, to evaluate safety of this combination, we compared the response of two cancer cell lines (A549 and NCI-H1703) and two normal cell lines (MDCK and nHCEC) to combination of doxorubicin and ZL-2201. We observed that after 24-hour washout of doxorubicin and ZL-2201, A549 and NCI-H1703 had IC_50_s of 18.76 and 7.48 μmol/L, respectively while both normal cell lines had IC_50_s greater than maximum concentration (44.4 μmol/L) used (Supplementary Fig. S5B). Next, we investigated the mechanisms driving synergy between doxorubicin and ZL-2201. Single-agent treatment with ZL-2201 or doxorubicin did not alter levels of two key phenotypic markers, phospho-histone H3 (mitotic index) and cleaved caspase 3 (apoptosis) after 72 hours. However, 72-hour combination with doxorubicin resulted in 2-fold and greater increases in both phospho-histone H3 and cleaved caspase 3 with increasing doses of ZL-2201 starting from 300 nmol/L (Supplementary Fig. S5C). To validate the increased mitotic index observed with combination of doxorubicin and ZL-2201, cell-cycle analysis was performed. ZL-2201 treatment alone did not alter the percentage of G_2_–M, although there was a dose-dependent increase in G_0_ population (Fig. 3D). In combination with the low 6 nmol/L dose of doxorubicin, there was a disitinct increase in G_2_–M cells at 100 and 300 nmol/L ZL-2201. Combination with ZL-2201 at doses of 1 and 3.6 μmol/L displayed decreases in G_2_–M with a proportional increase in the G_0_ population (Fig. 3D). To understand whether the increased mitotic index and apoptosis was linked to unrepaired DNA damage, we investigated the phosphorylation status of H2AX at Ser139. Similar to cell-cycle analysis, 72 hour treatment with ZL-2201 alone did not increase γH2AX levels. However, at both 24 and 72 hours, ZL-2201 treatment significantly increased γH2AX levels in the presence of 6 nmol/L doxorubicin (Fig. 3E). Cumulatively, these data illustrate that ZL-2201 robustly inhibits NHEJ-mediated repair of exogenous DNA damage resulting in increased G_2_–M arrest and apoptosis *in vitro*.

### ZL-2201 Exhibits *In Vivo* Antitumor Activity in Combination with IR and Doxorubicin

**FIGURE 4:**
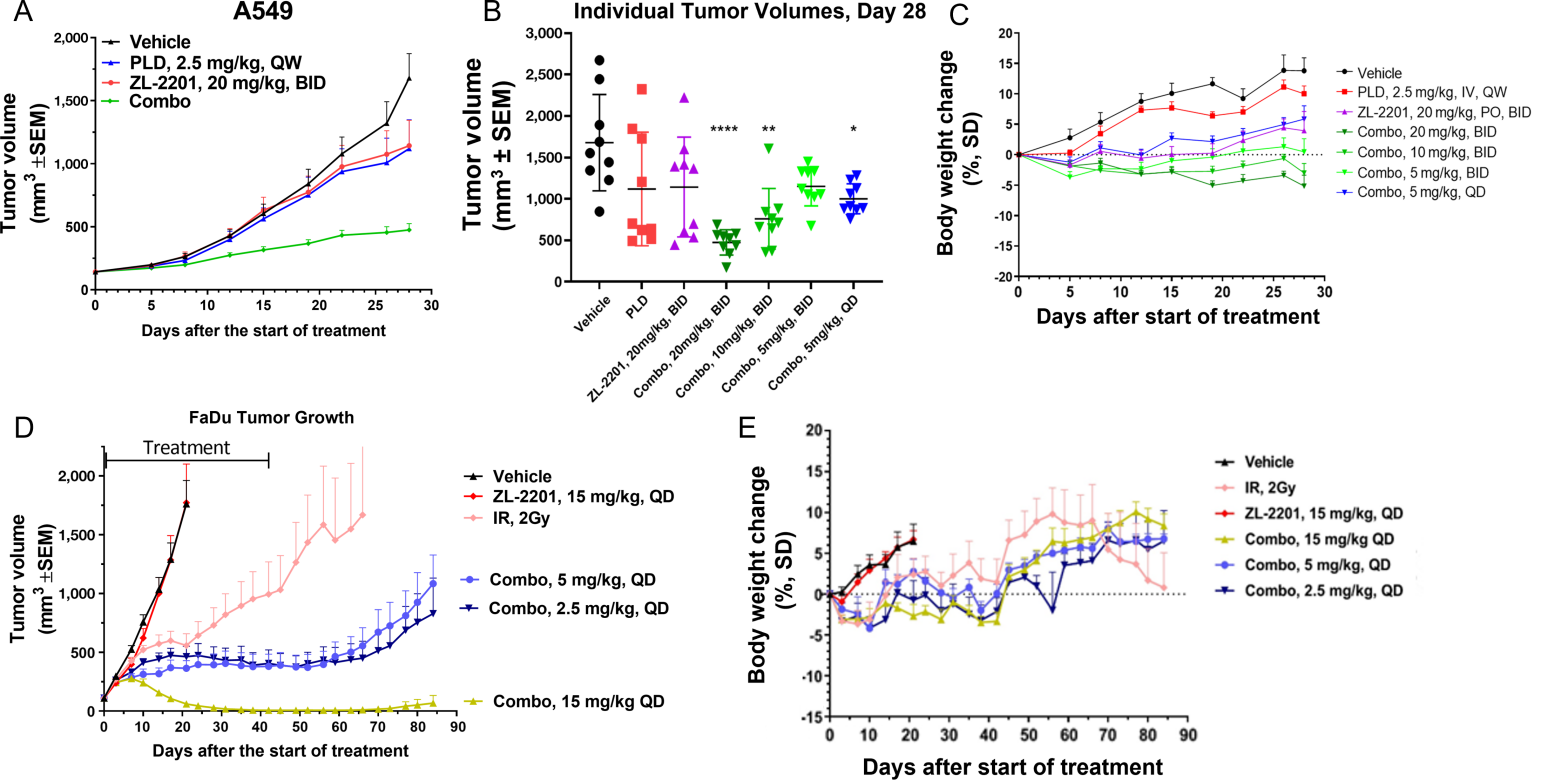
ZL-2201 significantly augments antitumor activity of DSB-inducing agents doxorubicin and IR. **A,** A549 xenografts established in female Balb/c nude mice (*n* = 8/group) were treated with vehicle, ZL-2201 (20 mg/kg; twice daily), PLD (2.5 mg/kg; every week) and ZL-2201 + PLD for 28 days. Average tumor volume is graphed. **B,** Individual tumor volumes for A549 xenografts treated with different doses of ZL-2201 (5, 10, 20 mg/kg twice daily) in combination with PLD. One-way ANOVA analysis of Vehicle versus Combo groups was performed. *, *P* = 0.035; **, *P* = 0.0011; ***, *P* < 0.0001. Kruskal–Wallis test: ZL-2201 versus Combo (*P* = 0.0403), PLD versus Combo (*P* = 0.0485). **C,** Body weight change for treatment arms shown in A. **D,** FaDu xenografts (*n* = 8/group) treated with Vehicle, ZL-2201(15 mg/kg; every day), IR (2 Gy), and ZL-2201 + IR for 40 days. Tumors were then allowed to grow without treatment for an additional 44 days. **E,** Mouse body weight changes for each treatment arm shown in **D**. QD, every day; QID, twice daily; QW, every week.

To further confirm the potential of ZL-2201 to augment DNA damage induced by DSB-inducing agents, we interrogated the *in vivo* combinatorial efficacy of ZL-2201 and doxorubicin or irradiation in ZL-2201–insensitive cell lines A549 and FaDu. The combination of ZL-2201 with PLD (34) was examined in mice bearing A549 xenografts. Single-agent treatment with either PLD (2.5 mg/kg, i.v., every week) or ZL-2201 (20 mg/kg, orally, twice daily) produced a moderate but nonsignificant reduction in tumor growth (TGI = 36.4% and 34.9%, *P* = 0.527 and 0.495 compared with vehicle control, respectively; Fig. 4A and B). However, the combination of PLD with ZL-2201 resulted in significant antitumor activity (TGI = 78.4%, *P* = 0.002) with no significant weight loss (Fig. 4C). Furthermore, we evaluated the therapeutic efficacy of ZL-2201 combined with ionizing radiotherapy, which induces DNA DSBs, in the FaDu human pharyngeal squamous cell carcinoma xenograft model. In this study, ZL-2201 was administered orally prior to focused X-ray irradiation of the subcutaneous tumor. Treatment with ZL-2201 alone at dose of 15 mg/kg did not produce significant antitumor activity (TGI = 25.5%; *P* = 0.243; Fig. 4D). Treatment with X-ray radiation alone (2 Gy) for 3 weeks resulted in significant antitumor activity (TGI = 63.3%, *P* < 0.001; Fig. 4D). When combined with focused irradiation, ZL-2201 treatment produced significant antitumor efficacy (TGI = 97.8% for 15 mg/kg once daily; *P* < 0.001) that exceeded that of irradiation alone (Fig. 4D). Importantly, coadministration of ZL-2201 significantly enhanced the antitumor efficacy of irradiation alone, even when ZL-2201 was administered at doses as low as 2.5 mg/kg once daily (Fig. 4D). No gross abnormalities were observed during the full necropsy of these animals. Although moderate body weight loss was observed for individual animals in the 5 and 15 mg/kg combination treatment groups, the impact was not marked enough to require removal of these animals from the study (Fig. 4E). Indeed, all these animals demonstrated resumption of normal body weight gain over time. Together, the results of this study indicate that ZL-2201 treatment can safely and effectively prolong the antitumor efficacy of DNA-damaging agents such as doxorubicin and IR.

### MS Screen Identifies New Phosphoproteins Altered by ZL-2201 Treatment

**FIGURE 5:**
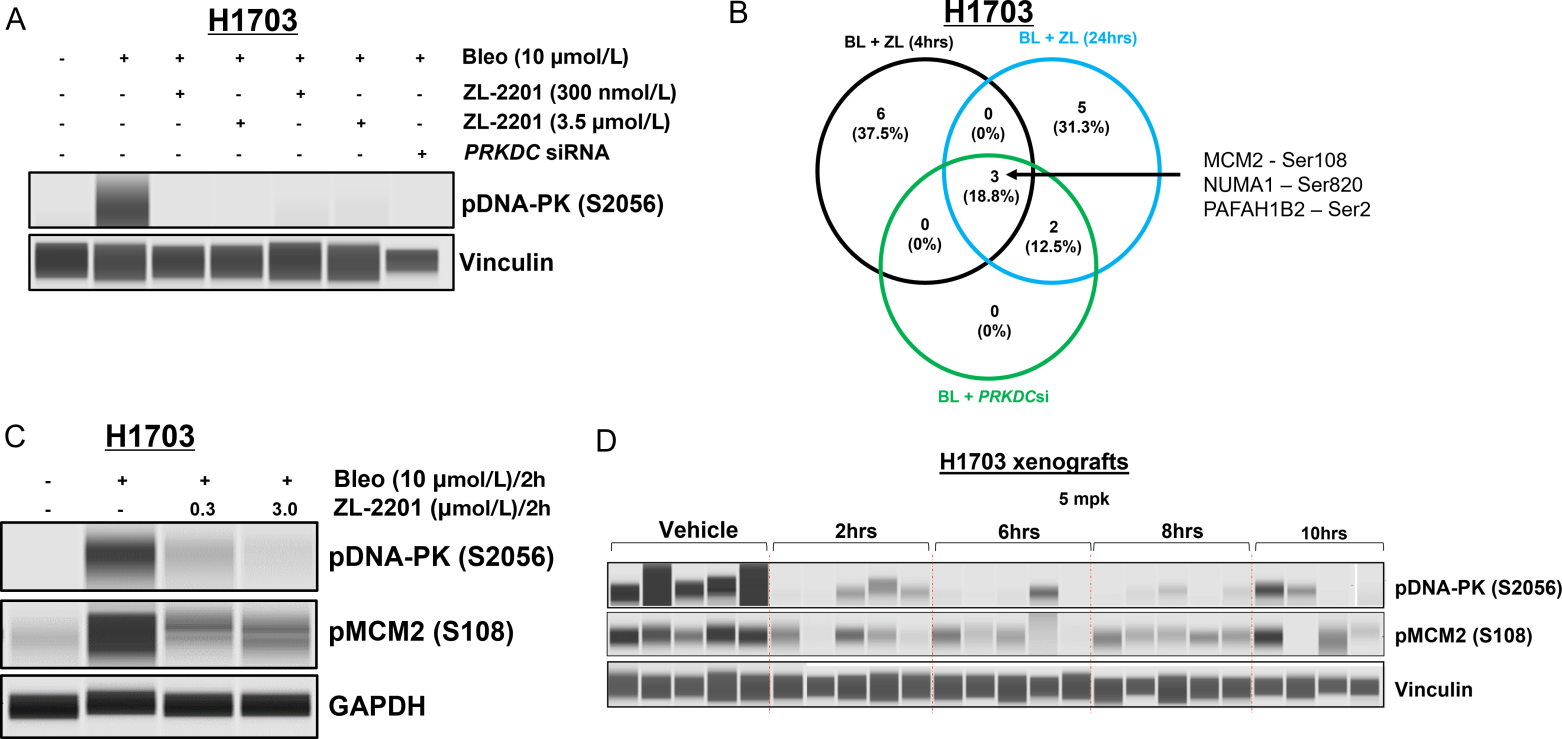
Phosphoproteomic screen identifies MCM2 Ser108 phosphorylation decreased by ZL-2201. **A,** Simple Western showing inhibition of pDNA-PK upon treatment with PRKDC siRNA, 300 nmol/L and 3.5 μmol/L of ZL-2201 ± 10 μmol/L bleomycin used in phospho-MS screen analysis. **B,** The Venn diagram of phosphoproteins with ATM and DNA-PK substrate motifs decreased with bleomycin + ZL-2201 and PRKDC siRNAs at the indicated timepoints. **C,** Simple Western validation showing inhibition of pMCM2 (S108) upon treatment with ZL-2201 in NCI-H1703 cells. **D,** Simple Western showing inhibition of pMCM2 (S108) upon treatment with ZL-2201 (5 mg/kg) in NCI-H1703 tumor xenografts over the course of 2–10 hours (*n* = 4–5).

To gain greater insight into the molecular mechanisms regulated by ZL-2201–mediated DNA-PK inhibition, we performed a proteomic MS screen to identify serine/threonine (Ser/Thr)-phosphorylated proteins modulated by DNA-PK inhibition. NCI-H1703 cells were treated with 300 nmol/L ZL-2201 for 4 and 24 hours, followed by 3 hours treatment with bleomycin (10 μmol/L). In addition, NCI-H1703 cells transfected with *PRKDC* siRNA for 48 hours followed by bleomycin treatment were included to identify DNA-PK specific proteomic hits. The phospho-DNA-PK levels of the samples were evaluated before MS analysis through immunoblotting (Fig. 5A). For this article, we focused our initial analysis on peptides with DNA-PK and ATM substrate motifs (XXSQXX). Similar to the immunoblot results, MS analysis revealed an increase in phospho-DNA-PK at Ser2612 (another autophosphorylation site on DNA-PK) following bleomycin treatment compared with the undetectable levels observed in the vehicle-treated samples. Next, we compared the phosphoproteomic changes between bleomycin + ZL2201 versus bleomycin alone. Interestingly, RPA Ser4/8 was not detected in this screen; however, we observed common significant decreases in phosphorylated MCM2 at Ser108, NUMA1 at Ser820, and PAFAH1B2 at Ser2 in samples treated for 4 and 24 hours with ZL-2201 and PRKDC siRNA in the presence of bleomycin (Fig. 5B). MCM2 is a key S-phase checkpoint protein and phosphorylation at Ser108 was previously shown to be increased by the DNA-damaging agent etoposide (35) and modulated by CDC7 and ATR (36). NUMA plays a critical role in mitotic spindle organization to support mitosis (37, 38). Because of limited antibody availability, we confirmed that ZL-2201 decreased phospho-MCM2 Ser108 levels across A549, NCI-H1703, and FaDu cell lines and NCI-H1703 mice xenografts (Fig. 5C and D; Supplementary Fig. S6A and S6B). These preliminary findings suggest that phospho-MCM2 (Ser108) can be used as a specific biomarker for DNA-PK inhibition, and may be a key contributor to the augmented G_2_–M arrest and apoptosis observed in combination with DSB-inducing agents. These hypotheses warrant further investigation in conjunction with analysis of the additional Ser/Thr kinases identified.

## Discussion

In this article, we characterize ZL-2201 as a potent DNA-PK inhibitor with remarkable selectivity compared with previously reported inhibitors. From a limited set of *in vitro* cell line models, ZL-2201 showed some promising single-agent activity (Fig. 1). Consistent with previous DNA-PK inhibitors that have been shown to potentiate the activity of IR, etoposide, and doxorubicin, ZL-2201 also showed strong synergy with etoposide and doxorubicin *in vitro* and significant antitumor activity with doxorubicin and IR *in vivo* (Figs. 3 and 4). Although ATM-deficient cells were more sensitive to ZL-2201 treatment, suggesting a synthetic lethal interaction (Fig. 1), the synergy with doxorubicin was not dependent on the ATM genetic background (Fig. 3). Finally, we identified two key cell-cycle proteins, MCM2 and NUMA1, that are dephosphorylated in the presence of bleomycin and ZL-2201, initiating future mechanistic understanding of DNA-PK inhibition.

The mechanism of action of different drugs and the magnitude of the treatment invoke the choice of the repair pathway upon DNA damage. Treatment with IR or doxorubicin in combination with ZL-2201 caused significant tumor regression *in vivo*, suggesting that the NHEJ pathway was the major choice of repair pathway in response to these DNA-damaging treatments and offered a clear combination benefit. Interestingly, DNA-PK autophosphorylation inhibition by ZL-2201 *in vivo* occurs as early as 30 minutes after treatment and continued in a dose-dependent duration of ZL-2201 in mice (Fig. 2). Because the half-life of ZL-2201 was observed to be less than 1 hour in mice (Table 2), twice a day dosing would likely be required for single-agent efficacy, particularly at lower dose levels. However, the daily dosing of ZL-2201 alongside IR also achieved significant tumor regression, even at low doses of ZL-2201, which suggests that NHEJ is engaged acutely upon radiation and can elicit rapid cancer cell death when NHEJ is inhibited, even transiently. Further studies with other relevant cancer mouse models will help to strengthen the combinational benefit of ZL-2201 with IR and doxorubicin. Taken together, these data support various dose regimens when ZL-2201 is combined with different DNA damage-inducing agents, which will provide flexibility in the clinic, depending on tolerance.

Genetic-based studies have described ATM as a synthetic lethal partner with DNA-PK (25–27). This is in line with the concept that loss of key upstream regulators of HR will render cells more dependent on NHEJ-mediated DNA repair. Using isogenic models, we observed increased sensitivity of ATM KO cells to ZL-2201 compared with their respective wild-type (WT) counterparts. Interestingly, when tested in cell lines with ATM mutations, the sensitivity window was smaller. One limitation of this study is the small number of cell lines tested; however, the initial findings suggest that complete loss of ATM may be necessary to observe single-agent activity. This is also consistent with the observations that lymphomas, which has the higher frequency of *ATM* deletions, display better response to DNA-PK inhibitors (27). In addition, the combination of ZL-2201 with DSB-inducing agent doxorubicin was independent of ATM status which indicates that DNA-damaging stress may be a stronger contributor to ZL-2201 sensitivity. This is in line with the observation by Zhou and colleagues, where DNA-PK was observed as a synthetic lethal partner in MYC-overexpressing cell lines (39). *MYC* overexpression results in increased DNA DSBs which suggests that *MYC* amplified models exhibit higher replication stress (40, 41). Further evaluation of single biomarker populations and replication stress signatures are warranted for further development of ZL-2201.

From the combination studies performed with ZL-2201, strong *in vitro* synergy was observed with doxorubicin and etoposide in both A549 and NCI-H1703 cell lines. This observation was concordant with the study performed with another DNA-PK inhibitor, M3814, which also displayed strong synergy with doxorubicin, etoposide, and bleomycin (19). While these results confirmed that strong DSB-inducing agents synergize with DNA-PK inhibition, we only observed *in vivo* tumor growth delay in the ZL-2201 and PLD *in vivo* combination study in A549 xenografts. Fok and colleagues observed similar tumor growth delay in A549 xenografts with combination of radiation and the DNA-PK inhibitor AZD7648, while tumor regression was observed with the same combination in H1299 xenografts (20). In the same article, combination of AZD7648 and PLD resulted in tumor regression of BT474 breast cancer xenograft model. Wise and colleagues also showed that the *in vivo* efficacy of M3814 and PLD combination was significantly greater in the p53-null SKOV3 model compared with the p53-WT A2870 model (42). With A549 being P53-WT and both H1299 and BT474 being P53-mutant, our observations confirm that p53 status may serve as a predictor of improved combination efficacy with ZL-2201. This can be further corroborated by our observation of tumor regression with the combination of radiation and ZL-2201 in the p53-mutant FaDu model. Further *in vivo* profiling across multiple indications and bioinformatic analysis will strengthen the validity of this hypothesis for clinical development.

A phosphoproteomic MS screen was performed in NCI-H1703 cells treated with ZL-2201 and the DBS-inducing insult bleomycin. This study was used to determine the mechanistic pathways engaged and identify potential biomarkers of target engagement. For this article, initial focus was placed on proteins with DNA-PK/ATM substrate motifs to identify proteins directly affected by ZL-2201. MCM2 (Ser108), NUMA1(Ser820) and PAFAH1B2(Ser2) were three common proteins found to exhibit decreased phosphorylation with ZL-2201 or PRKDC RNAi + Bleomycin. One limitation of this study was the inability to validate NUMA1 and PAFAH1B2 phosphorylation due to lack of commercially available antibodies. Ser108 on MCM2 has been shown to be partially regulated by the S-phase kinase Cdc7(43) and phosphorylated by ATM and ATR (43). In line with publications, Ser108 phosphorylation has also been shown to robustly increase with DSB-inducing insults etoposide, hydroxyurea, and radiation (43). Decreased Ser108 phosphorylation was observed in both ATM-proficient (A549 and FaDu) and ATM-deficient cell line (NCI-H1703) suggesting that ATM and DNA-PK may play redundant roles in phosphorylating MCM2 and warrants further investigation. NUMA1 plays a critical role in mitotic spindle assembly and Ser820 is found in the coil-coil domain of NUMA1 which is required for spindle pulling force generation and inhibition of chromatin binding during anaphase for formation of well-shaped nucleus (37, 44). Zl-2201 combined with doxorubicin increased G_2_–M accumulation and the mitotic index marker, phospho-histone H3 which correlated with increased DNA damage (γH2AX) and apoptosis (cleaved caspase 3). These observations speculate that molecular mechanisms obstructing mitosis may be key to ZL-2201 activity in combination with DSB-inducing agents. Furthermore, Moreno and colleagues illustrated that bleomycin and IR induced Ser395 phosphorylation of NUMA1 which prevents the accumulation of 53BP1 foci at DNA breaks (45). Fok and colleagues observed increased 53BP1 foci with combination of AZD7648 and doxorubicin (20), therefore it is possible that decreased NUMA1 phosphorylation by DNA-PK inhibition may contribute to this accumulation of 53BP1 foci and consequent DNA damage. These promising observations warrant further validation of the proteomic hits including those that do not have ATM/DNA-PK substrate motifs in hope of understanding the molecular underpinnings of ZL-2201 and identification of novel targets.

The continued interest in developing more selective and potent DNA-PK inhibitors is evident from the well-established role of DNA-PK in the NHEJ pathway and in the response to DDR-inducing chemotherapeutics and IR therapies. Here, we report the development of a more specific small-molecule inhibitor of DNA-PK, ZL-2201. Several lines of data suggest the dependency of cancer cells on the HR or NHEJ pathways in response to the DNA DSBs in numerous malignancies. We showed sensitivity of the cancer cells to ZL-2201 with ATM deficiency; however, the synergy of ZL-2201 with topoisomerase II inhibitors was not ATM dependent. Therefore, ATM cannot be used as a biomarker for ZL-2201 efficacy. In addition, the study does not follow a specific histology of cancer, instead, evidence provided here indicates the therapeutic potential of ZL-2201 in the treatment of various tumor types as a sensitizer to DNA DSB-inducing agents. As such, ZL-2201 is anticipated to advance to the clinic.

## Supplementary Material

Supplementary Methods Supplementary Methods

Table S1 ZL-2201 anti-proliferative IC50s and ATM mutational and VAF status

Figure S1 Synthesis of ZL-2201

Figure S2 Cellular efficacy of ZL-2201 across cancer cell lines

Figure S3 Kinetics of Bleomycin-induced DNA-PK phosphorylation by ZL-2201

Figure S4 ZL-2201 treatment exhibits potent antitumor efficacy in vivo.

Figure S5 Phenotypic effects of combining ZL-2201 and Doxorubicin

Figure S6 ZL-2201 reduces DNA damage-induced MCM2 phosphorylation (Ser108).
