# Supplementary material for: Discovery and Characterization of ZL-2201, a Potent, Highly Selective, and Orally Bioavailable Small-molecule DNA-PK Inhibitor
Source: Cancer Res Commun. 2023 Sep 1;3(9):1731–42. doi: 10.1158/2767-9764.CRC-23-0304 (PMC10473160; doi:10.1158/2767-9764.CRC-23-0304)
Supplement: Supplementary Methods [file crc-23-0304-s01.docx]

**Supplementary Information**

**Discovery and characterization of ZL-2201, a potent, highly-selective, and orally bioavailable small-molecule DNA-PK inhibitor**

Shruti Lal^1^, Neil E. Bhola^1^, Bee-Chun Sun^1^, Yuping Chen^1^, Tom Huang^1^, Vivian Morton^1^, Kevin X. Chen^2^, Shanghua Xia^2^, Haoyu Zhang^2^, Nehal S. Parikh^1^, Qiuping Ye^1^, O. Petter Veiby^1^, David I. Bellovin^1^, and Yuhua Ji^1^

^1^Discovery, Zai Lab (US) LLC, 1440 O’Brien Dr., Suite A & C, Menlo Park, USA

^2^Wuxi Apptec, 288 Middle Fute Road, 2-226 Building 1, Pudong New District, Shanghai, China

Supplementary Methods

Synthesis of ZL-2201

Compound 1b: At 0 °C, a solution of sodium nitrite (2.28 g, 33 mmol, 1.1 *eq*) in water (4.5 mL) was slowly added to a mixed solution of compound **1a** (4.49 g, 30 mmol, 1 *eq*, hydrochloride) in acetic acid (50 mL) and water (18 mL). Then, the reaction mixture was stirred at 20 °C for 3 hours. After reaction completion, the mixture was diluted with water (50 mL), extracted with 150 mL of ethyl acetate (3x50 mL), and the organic phase was washed with saturated brine (30 mL), dried over anhydrous sodium sulfate, filtered. The filtrate was concentrated under reduced pressure and purified by column chromatography using ethyl acetate and petroleum ether as eluent (from 0:1 to 1:1 ratio) to obtain compound **1b**. MS: *m/z*. 143.0 [M+H]^+^.

Compound 1c: To a solution of compound **1b** (3.70 g, 26 mmol, 1 *eq*) in methanol (20 mL) at 0°C, zinc powder (6.80 g, 104 mmol, 4 *eq*) and acetic acid (20 mL) were added sequentially. Then the reaction mixture was stirred at 20°C for 4 hours. After the reaction was completed, the mixture was filtered through celite and washed with ethyl acetate (200 mL), and the filtrate was concentrated under reduced pressure to obtain a crude product of compound **1c**.

Compound 1e: At 0 °C, compound **1c** (4.89 g, 26 mmol, 1 *eq*) and triethylamine (13.15 g, 130 mmol, 5 *eq*, 18.09 mL) were added sequentially to a solution of 2,4-dichloro-5-nitropyrimidine (**1d**, 0.09 g, 52 mmol, 2 *eq*) in dioxane (150 mL). Then the reaction mixture was kept under stirring at 20 °C for 5 hours. After reaction completion, the mixture was diluted with water (100 mL), extracted with 300 mL of ethyl acetate (3x100 mL), and the organic phase was washed with saturated brine (50 mL), dried over anhydrous sodium sulfate, filtered, and the filtrate was concentrated under reduced pressure and purified by column chromatography using ethyl acetate/petroleum ether as eluent (0-50%) to obtain compound **1e**. MS: *m/z* 285.9 [M+H]^+^.

^1^H NMR (400 MHz, CDCl_3_) δ ppm 9.05 (s, 1H), 8.97 (br s, 1H), 4.43 (br dd, *J*=4.44, 2.06 Hz, 2H), 2.94-3.06 (m, 4H), 2.19-2.28 (m, 2H), 1.90-2.03 (m, 2H).

Compound 1f: To a solution of compound **1e** (2.43 g, 8.5 mmol, 1 *eq*) in ethanol (120 mL) and water (30 mL), iron powder (2.37 g, 42.5 mmol, 5 *eq*) and ammonium chloride (2.27 g, 42.5 mmol, 5 *eq*) were added sequentially. Then the mixture was heated at 75 °C under stirring for 3 hours. After reaction completion, the mixture was cooled to room temperature and diluted with ethyl acetate (200 mL), filtered through celite and concentrated under reduced pressure to obtain a crude product of compound **1f**. MS: *m/z* 256.0 [M+H]^+^.

Compound 1g: To a solution of compound **1f** (2.17 g, 8.5 mmol, 1 *eq*) in acetonitrile (30 mL), *N,N'*-carbonyldiimidazole (2.76 g, 17 mmol, 2 *eq*) was added. Then the reaction mixture was stirred at 80 °C for 2 hours, followed by work-up. After reaction completion, the mixtire was concentrated under reduced pressure and purified by column chromatography using ethyl acetate/petroleum ethe as eluent (0-100%) to obtain compound **1g**. MS: *m/z* 281.9 [M+H]^+^.

^1^H NMR (400 MHz, DMSO-*d*_6_) δ ppm 11.61 (br s, 1H), 8.12 (s, 1H), 4.38 (br d, *J*=2.01 Hz, 2H), 3.73 (dd, *J*=9.91, 1.63 Hz, 2H), 2.81 (d, *J*=9.54 Hz, 2H), 1.99-2.09 (m, 2H), 1.78-1.87 (m, 2H).

Compound 1h: Cesium carbonate (2.15 g, 6.6 mmol, 1.5 *eq*) and iodomethane (780 mg, 5.5 mmol, 1.25 *eq*) were added sequentially to a solution of compound **1g** (1.24 g, 4.4 mmol, 1 *eq*) in *N,N*-dimethylformamide (40 mL). After addition, the mixture was stirred at room temperature for 4 hours. The reaction mixture was then diluted with water (50 mL), extracted with 180 mL of ethyl acetate (3x60 mL), and the organic phase was washed with saturated brine (50 mL), dried over anhydrous sodium sulfate, filtered, and the filtrate was concentrated under reduced pressure and purified by column chromatography using ethyl acetate/petroleum ether as eluent (0-80%) to obtain compound **1h**. MS: *m/z* 295.9 [M+H]^+^.

^1^H NMR (400 MHz, CDCl_3_) δ ppm 7.98-8.05 (m, 1H), 4.45 (br d, *J*=2.25 Hz, 2H), 3.99 (dd, *J*=9.69, 1.81 Hz, 2H), 3.41 (s, 3H), 2.80 (br d, *J*=9.51 Hz, 2H), 2.23-2.31 (m, 2H), 1.94-2.04 (m, 2H).

Compound 1: Compound **1h** (502.7 mg, 1.7 mmol, 1 *eq*), 7-methyl-[1,2,4]triazolo[1,5-a]pyridin-6-amine (**1i**, 201.5 mg, 1.36 mmol, 0.8 *eq*), methanesulfonato(2-dicyclohexylphosphino-3,6-dimethoxy-2',4',6'-tri-i-propyl-1,1'-biphenyl)(2'-amino-1,1'-biphenyl-2-yl)palladium(II) (231.2 mg, 255µmol, 0.15 *eq*) and cesium carbonate (830.8 mg, 2.55 mmol, 1.5 *eq*) were placed in a reaction flask, and the reaction flask was vacuumized and replaced with nitrogen three times, then anhydrous dioxane (30 mL) was added. The reaction mixture was stirred at 100 °C for 3 hours. After the reaction was completed, the mixture was filtered through celite and concentrated under reduced pressure to obtain a crude product, then the crude product was purified by column chromatography (methanol/dichloromethane: 0-10 %) and slurring in a mixture of dichloromethane/ethyl acetate (1.5 mL/3 mL) at 25 °C for 15 min, to obtain compound **1 (ZL-2201)**. MS: *m/z* 408.2 [M+H]^+^.

^1^H NMR (400 MHz, CDCl_3_) δ ppm 9.87 (s, 1H), 8.26 (s, 1H), 7.91 (s, 1H), 7.57 (s, 1H), 6.76 (s, 1H), 4.48 (br d, *J*=2.25 Hz, 2H), 4.04 (dd, *J*=9.76, 1.88 Hz, 2H), 3.40 (s, 3H), 2.85 (d, *J*=9.51 Hz, 2H), 2.53 (s, 3H) 2.29-2.37 (m, 2H), 2.00-2.10 (m, 2H).

**Hepatocyte stability and plasma protein binding assay:** For hepatocyte stability assay, ZL-2201 was incubated at 1 µM in 0.5 million per mL hepatocyte (BioIVT) from preclinical species and human at 37°C with humidity and CO_2_ control, samples were taken at various time points for up to 2 hours. For plasma protein binding, ZL-2201 was spiked to plasma which was adjusted pH to 7.4 across species, respectively, at a final concentration of 1 µM. The spiked plasma samples were incubated at 37ºC for 15 min, then centrifuged at 120,000 rpm, 37ºC for 3 hours in an ultracentrifuge (Beckman Coulter). The supernatants were purified with protein precipitation method and analyzed using LC-MS/MS method for ZL-2201. Samples of both assays were analyzed using a LC-MS/MS system (Applied Biosystems) with standard curves.

**Compounds/Reagents:**

| **Compounds** | **Source** | **Catalog Number** |
| --- | --- | --- |
| ZL-2201 | Zai Lab |  |
| Dimethylsulfoxide (DMSO) | ATCC | 50-188-278FP |
| Docetaxel | MilliporeSigma | 11-101-3167 |
| Gemcitabine | MilliporeSigma | 50-459-40001 |
| 5-Fluorouracil | Tocris Bioscience | 32-575-0 |
| Topotecan | Selleck Chemical | 50-194-8345 |
| Camptothecin | TCI AmericA | C1495100MG |
| Irinotecan | TCI AmericA | I0714100MG |
| Cisplatin | MilliporeSigma | 23-212-050MG |
| Doxorubicin | Thermo Scientific | AAJ64000MF |
| Niraparib | Selleck Chemical | NC1961274 |
| Etoposide | Thermo Scientific | AAJ63651MC |
| Temozolomide | TCI America | T2744500MG |
