# Supplementary figures and images for: Discovery and Characterization of ZL-2201, a Potent, Highly Selective, and Orally Bioavailable Small-molecule DNA-PK Inhibitor
Source: Cancer Res Commun. 2023 Sep 1;3(9):1731–42. doi: 10.1158/2767-9764.CRC-23-0304 (PMC10473160; doi:10.1158/2767-9764.CRC-23-0304)

## Slide 1
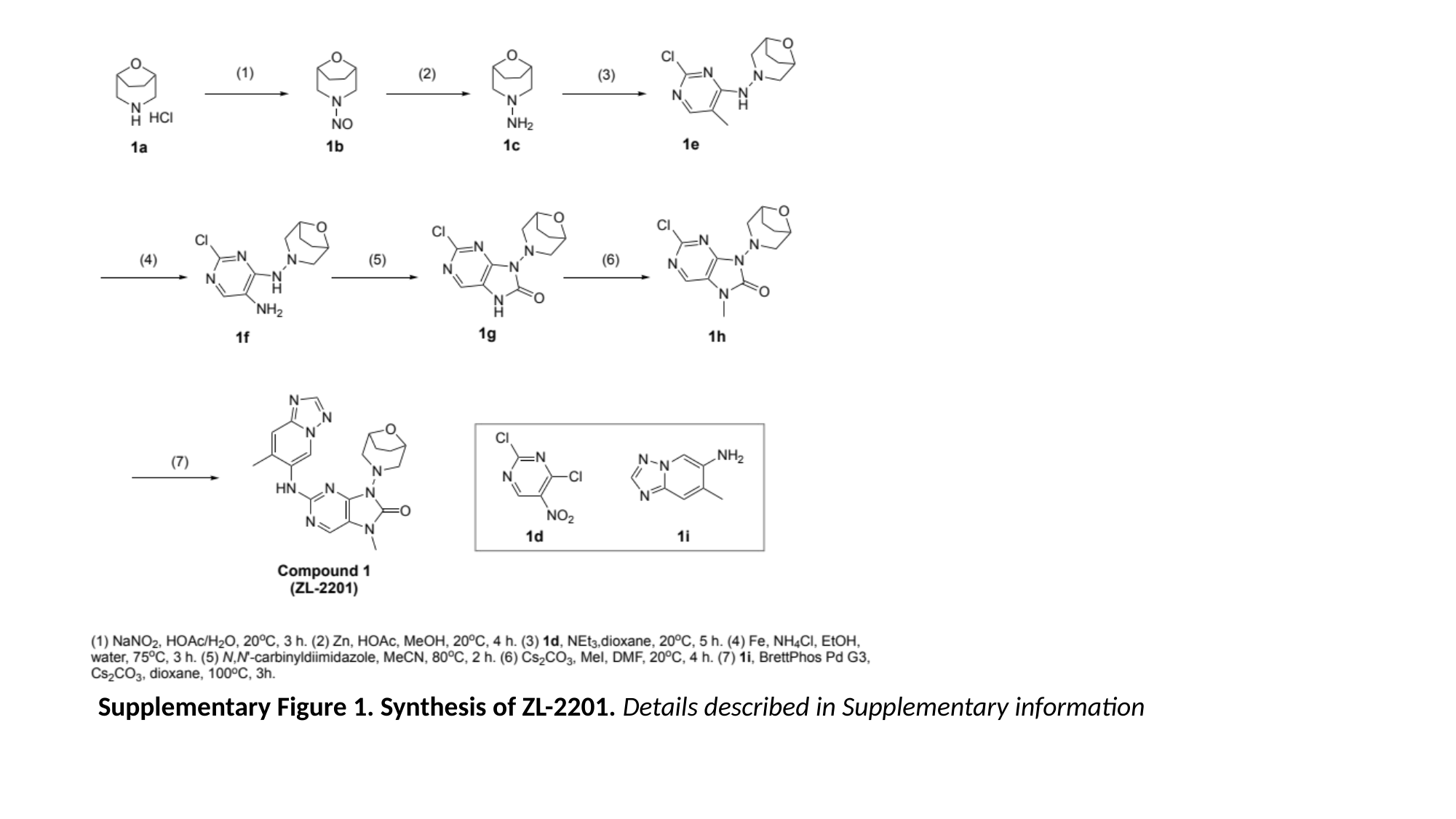

Supplementary Figure 1. Synthesis of ZL-2201. Details described in Supplementary information

Supplement: Figure S1 — Synthesis of ZL-2201 [file crc-23-0304-s03.pptx]
