# Supplementary material for: Discovery and Characterization of ZL-2201, a Potent, Highly Selective, and Orally Bioavailable Small-molecule DNA-PK Inhibitor
Source: Cancer Res Commun. 2023 Sep 1;3(9):1731–42. doi: 10.1158/2767-9764.CRC-23-0304 (PMC10473160; doi:10.1158/2767-9764.CRC-23-0304)
Supplement: Figure S2 — Cellular efficacy of ZL-2201 across cancer cell lines [file crc-23-0304-s04.pptx]

## Slide 1
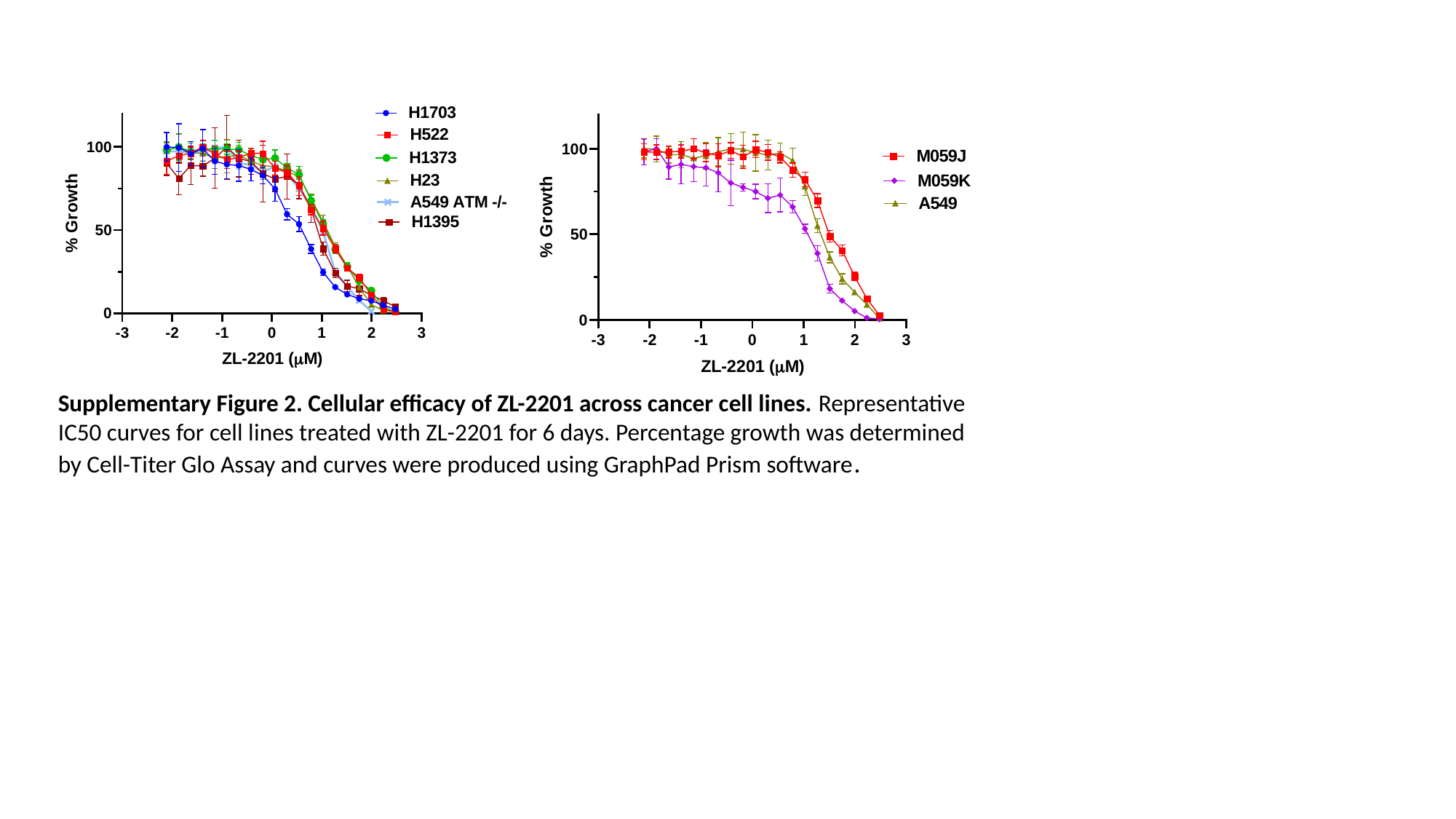

Supplementary Figure 2. Cellular efficacy of ZL-2201 across cancer cell lines. Representative IC50 curves for cell lines treated with ZL-2201 for 6 days. Percentage growth was determined by Cell-Titer Glo Assay and curves were produced using GraphPad Prism software.
