# Supplementary material for: Discovery and Characterization of ZL-2201, a Potent, Highly Selective, and Orally Bioavailable Small-molecule DNA-PK Inhibitor
Source: Cancer Res Commun. 2023 Sep 1;3(9):1731–42. doi: 10.1158/2767-9764.CRC-23-0304 (PMC10473160; doi:10.1158/2767-9764.CRC-23-0304)
Supplement: Figure S3 — Kinetics of Bleomycin-induced DNA-PK phosphorylation by ZL-2201 [file crc-23-0304-s05.pptx]

## Slide 1
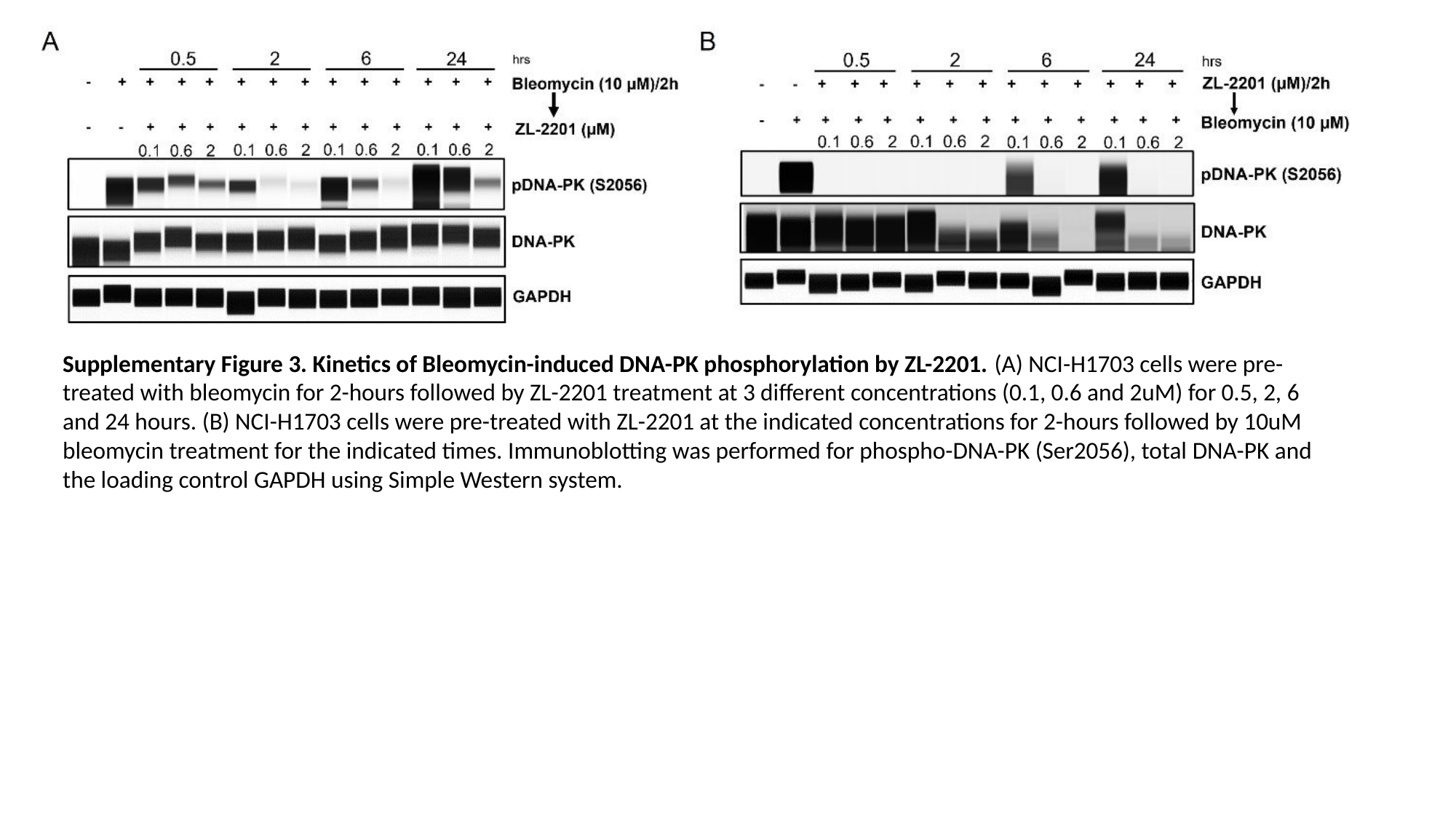

Supplementary Figure 3. Kinetics of Bleomycin-induced DNA-PK phosphorylation by ZL-2201. (A) NCI-H1703 cells were pre-treated with bleomycin for 2-hours followed by ZL-2201 treatment at 3 different concentrations (0.1, 0.6 and 2uM) for 0.5, 2, 6 and 24 hours. (B) NCI-H1703 cells were pre-treated with ZL-2201 at the indicated concentrations for 2-hours followed by 10uM bleomycin treatment for the indicated times. Immunoblotting was performed for phospho-DNA-PK (Ser2056), total DNA-PK and the loading control GAPDH using Simple Western system.
