# Supplementary material for: Discovery and Characterization of ZL-2201, a Potent, Highly Selective, and Orally Bioavailable Small-molecule DNA-PK Inhibitor
Source: Cancer Res Commun. 2023 Sep 1;3(9):1731–42. doi: 10.1158/2767-9764.CRC-23-0304 (PMC10473160; doi:10.1158/2767-9764.CRC-23-0304)
Supplement: Figure S4 — ZL-2201 treatment exhibits potent antitumor efficacy in vivo. [file crc-23-0304-s06.pptx]

## Slide 1
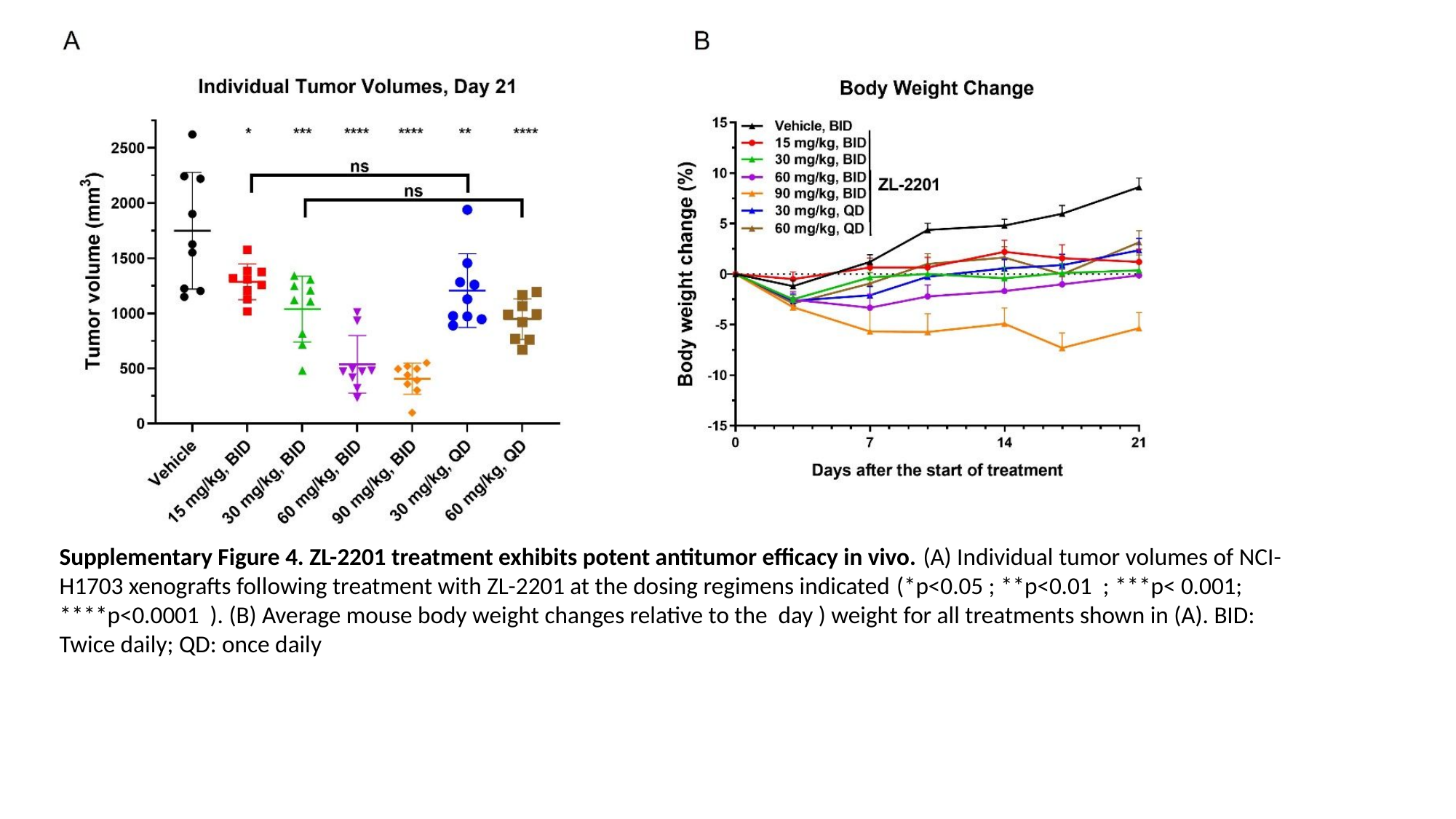

Supplementary Figure 4. ZL-2201 treatment exhibits potent antitumor efficacy in vivo. (A) Individual tumor volumes of NCI-H1703 xenografts following treatment with ZL-2201 at the dosing regimens indicated (*p<0.05 ; **p<0.01 ; ***p< 0.001; ****p<0.0001 ). (B) Average mouse body weight changes relative to the day ) weight for all treatments shown in (A). BID: Twice daily; QD: once daily
