# Supplementary material for: Discovery and Characterization of ZL-2201, a Potent, Highly Selective, and Orally Bioavailable Small-molecule DNA-PK Inhibitor
Source: Cancer Res Commun. 2023 Sep 1;3(9):1731–42. doi: 10.1158/2767-9764.CRC-23-0304 (PMC10473160; doi:10.1158/2767-9764.CRC-23-0304)
Supplement: Figure S5 — Phenotypic effects of combining ZL-2201 and Doxorubicin [file crc-23-0304-s07.pptx]

## Slide 1
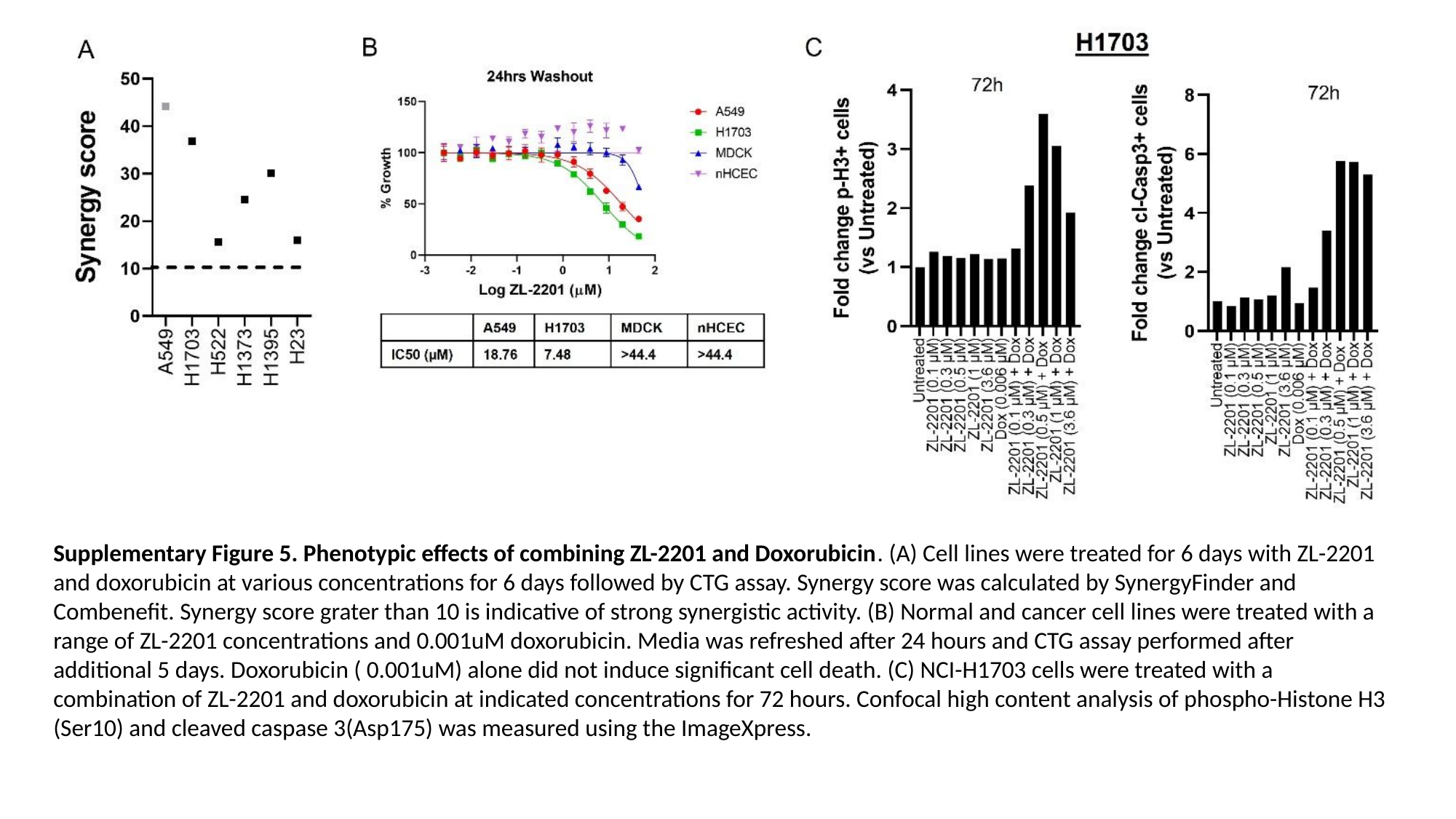

Supplementary Figure 5. Phenotypic effects of combining ZL-2201 and Doxorubicin. (A) Cell lines were treated for 6 days with ZL-2201 and doxorubicin at various concentrations for 6 days followed by CTG assay. Synergy score was calculated by SynergyFinder and Combenefit. Synergy score grater than 10 is indicative of strong synergistic activity. (B) Normal and cancer cell lines were treated with a range of ZL-2201 concentrations and 0.001uM doxorubicin. Media was refreshed after 24 hours and CTG assay performed after additional 5 days. Doxorubicin ( 0.001uM) alone did not induce significant cell death. (C) NCI-H1703 cells were treated with a combination of ZL-2201 and doxorubicin at indicated concentrations for 72 hours. Confocal high content analysis of phospho-Histone H3 (Ser10) and cleaved caspase 3(Asp175) was measured using the ImageXpress.
