# Supplementary material for: Discovery and Characterization of ZL-2201, a Potent, Highly Selective, and Orally Bioavailable Small-molecule DNA-PK Inhibitor
Source: Cancer Res Commun. 2023 Sep 1;3(9):1731–42. doi: 10.1158/2767-9764.CRC-23-0304 (PMC10473160; doi:10.1158/2767-9764.CRC-23-0304)
Supplement: Figure S6 — ZL-2201 reduces DNA damage-induced MCM2 phosphorylation (Ser108). [file crc-23-0304-s08.pptx]

## Slide 1
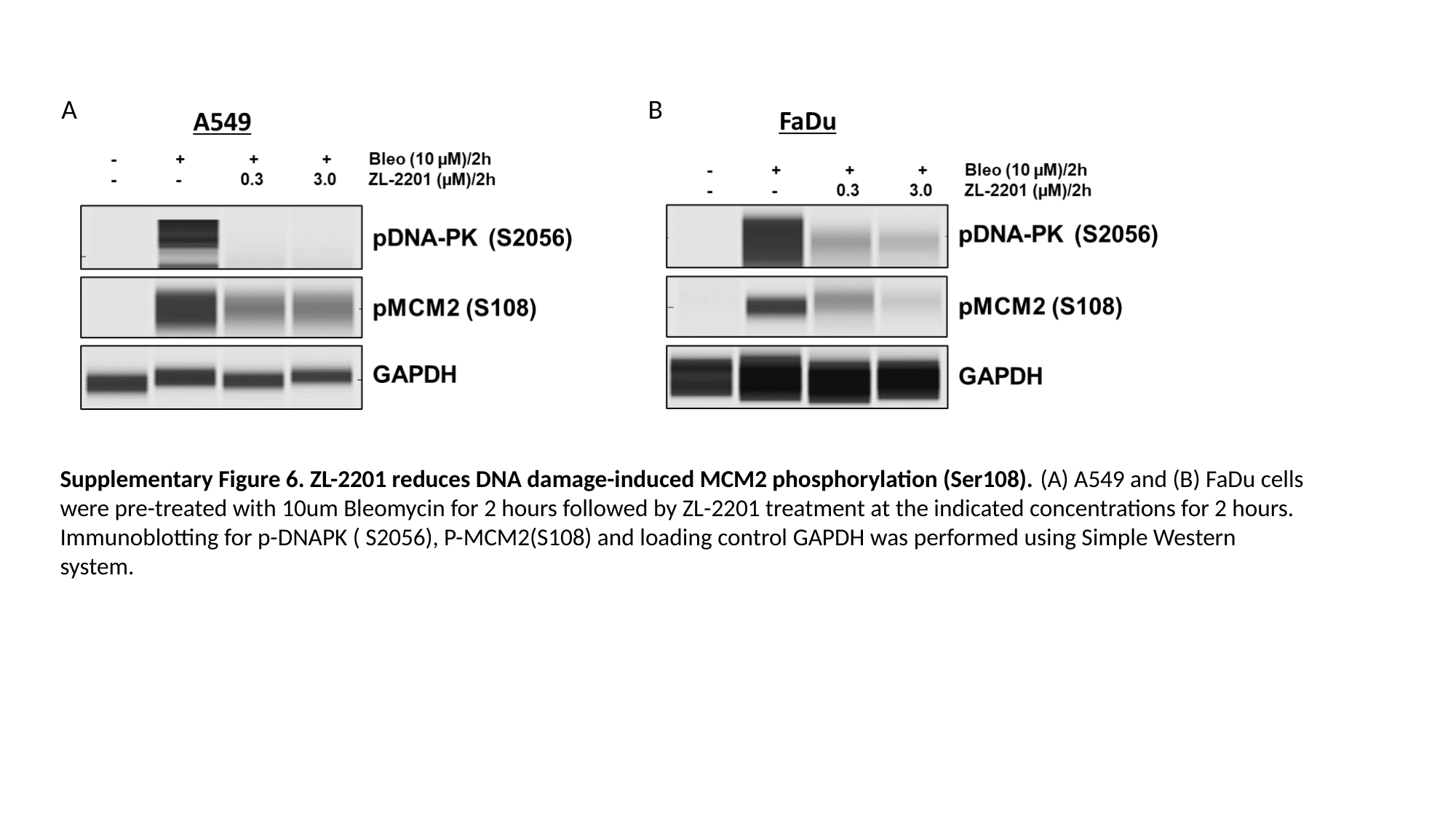

A
B
Supplementary Figure 6. ZL-2201 reduces DNA damage-induced MCM2 phosphorylation (Ser108). (A) A549 and (B) FaDu cells were pre-treated with 10um Bleomycin for 2 hours followed by ZL-2201 treatment at the indicated concentrations for 2 hours. Immunoblotting for p-DNAPK ( S2056), P-MCM2(S108) and loading control GAPDH was performed using Simple Western system.
